# Factors associated with the discontinuation of modern methods of contraception in the low income areas of Sukh Initiative Karachi: A community-based case control study

**DOI:** 10.1371/journal.pone.0218952

**Published:** 2019-07-03

**Authors:** Rozina Thobani, Saleem Jessani, Iqbal Azam, Sayyeda Reza, Neelofar Sami, Shafquat Rozi, Farina Abrejo, Sarah Saleem

**Affiliations:** Department of Community Health Sciences, Aga Khan University, Karachi, Pakistan; Anglia Ruskin University, UNITED KINGDOM

## Abstract

**Introduction:**

Discontinuation of a contraceptive method soon after its initiation is becoming a public health problem in Low middle income countries and may result in unintended pregnancy and related unwanted consequences. A better understanding of factors behind discontinuation of a modern method would help in designing interventions to continue its use till desired spacing goals are achieved.

**Objective:**

To determine factors associated with the discontinuation of modern contraceptive methods within six months of its use compared to continued use of modern method for at least six months in low-income areas of Karachi, Pakistan.

**Methods:**

A community-based case-control study was conducted in low-income areas of Karachi. Cases were 137 users who discontinued a modern contraceptive method within 6 months of initiation and were not using any method at the time of interview, while controls were 276 continuous users of modern method for at least last six months from the time of interview. Information was collected by using a structured questionnaire. Applied logistic regression was used to identify the associated factors for discontinuation.

**Results:**

The mean ages of discontinued and continued users were 29.3±5.3 years and 29.2±5.4 years respectively. A larger proportion of the discontinued users had no formal education (43.8%) as compared to the continued users (27.9%). The factors associated with discontinuation of a modern method of contraception were belonging to Sindhi ethnicity [OR: 2.54, 95%CI 1.16–5.57], experiencing side effects [OR: 15.12; 95% CI 7.50–30.51], difficulty in accessing contraceptives by themselves [OR: 0.40, 95%CI 0.19–0.83] and difficulty in reaching clinics for management of the side effects [OR: 4.10, 95%CI 2.38–7.05]. Moreover, women having support from the husband for contraceptive use were less likely to discontinue the method [OR: 0.58, 95% CI 0.34–0.98].

**Conclusions:**

Sindhi ethnicity and side effects of modern methods of contraception were identified as major factors for discontinuation in low-income populations. Similarly, women who had difficulty in travelling to reach clinics for treatment also contributed to discontinuation. Furthermore, women using long acting methods and those supported by their husbands were less likely to discontinue the contraceptive methods. Findings emphasize a need to focus on Sindhi ethnicity and trainings of service providers on management of side effects and provision of high quality of services.

## Introduction

WHO (World Health Organization) defines contraceptive prevalence rate as the percentage of women who are currently using, or whose sexual partner is currently using, at least one method of contraception, regardless of the method used [1]. It is usually reported for married or in-union women aged 15 to 49 years. Globally, the contraceptive use has increased from 55% in 1990 to 64% in 2015. However, about 12% women are still there around world who want to delay or avoid pregnancy are not using any method of contraception and this level is much higher in LMICs (Low and middle income countries), especially in sub-Saharan African countries at 24% [2].

The contraceptive discontinuation is the phenomena of starting a contraceptive method and then stopping it within one year of its use. Nearly, 20–50% of reversible modern methods users discontinue using a method during the first 12 months of initiating it and another 7–27% stop using a contraceptive method for reasons linked to the quality of service, non-availability of method of their choice, stock outs, and ineffective referral system [3]. The discontinuation of contraceptive methods occurs more with the methods that can be passively discontinued, such as condoms, injectable, pills and traditional methods as compared to the methods needing active discontinuation, such as the implants and intrauterine devices (IUD) [4–12]. In LMICs discontinuation of contraception often leads to unintended pregnancies and reduces the impact of family planning programs [11, 13].

Women may abandon contraception because they want a child or they get pregnant while using a method; they may experience adverse reactions to their chosen method; or they or their partners may find the method difficult or unpleasant to use, want a break from using it, or fear that continued use will lead to the side effects [14–15]. Access to care is another important reason for discontinuing a method [16].

Pakistan is the world’s sixth most populous country with a history of slow fertility decline in comparison to other Asian countries [17]. The high fertility rate and low use of contraception contributes to poor reproductive health indicators for women and high neonatal mortality [18]. Moreover, discontinuation of contraception has been found to be one of the reasons for a large number of induced abortions in Pakistan [19]. In 2002, abortion rate for Pakistan was estimated as 27 per 1000 women and in 2012, the rate of abortion almost doubled to 50 per 1000 women highlighting the importance of continued use of contraceptive methods to prevent unintended pregnancies [20].

According to Pakistan Demographic and Health Survey (PDHS 2012–2013), the ever use of modern contraceptive methods is at 54% while the current use is at 35%, which indicates that women who had started using modern contraceptives were giving them up and not returning back as indicated by the large gap between numbers of the ever and current users [21]. Twenty-two percent of women discontinued using contraceptives due to their side effects; 16% of women became pregnant while using contraceptives; 2% discontinued because of lack of access to contraceptives; and 3% discontinued due to disapproval from husbands [21].

With this backdrop, we hypothesized that women who discontinue to use a modern method of contraception are more likely to experience side effects, lack husband’s support, have difficulty in accessing contraceptives, have low education status, and have less desirous health seeking behavior as compared to women who continued to use a modern contraceptive method for at least six months.

## Methods

A community-based case-control study was conducted at the Sukh Initiative’s intervention sites in the urban slum areas. The ‘Sukh Initiative’ aims to provide quality family planning services to the community at their doorsteps and through public and private health care facilities. It is implemented by Aman Health Care Services, Aman Foundation Karachi, in ten underserved areas of Karachi, Pakistan, located in Bin Qasim, Korangi, Landhi and Malir towns and covers a population of approximately 1 million people. Present study targeted married women of reproductive age who were already exposed to the family planning messages and services through this project for last two years.

Discontinued users (Cases) were women who had ever used a modern method of contraception within last one year, and discontinued its use within six months of its initiation and were not using any method at the time of enrollment in the study. Continued users (Controls) were the women who were using a modern method of contraception for at least last six months without disruption at the time of enrollment in the study.

Modern methods were defined as IUD, injectable, implant, pills, condoms, tubal ligation, male sterilization and Lactational Amenorrhea (LAM). Socioeconomic status was defined based on the ownership status of house, rooms in house, and household assets (24 items including assets), and income.

**Fig 1:**
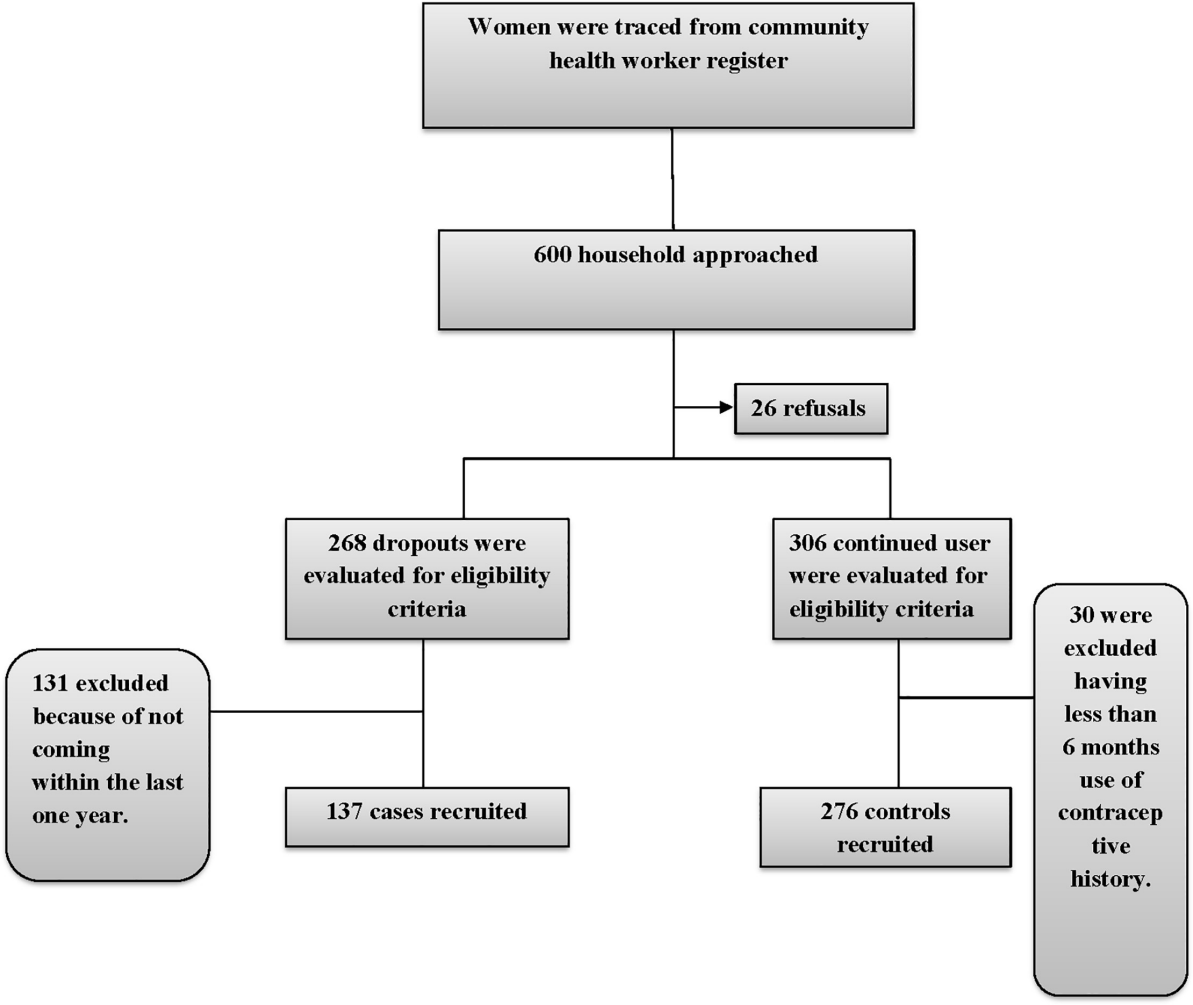
The flow chart of recruitment of participants.

The discontinued users and continued users were identified through reviewing the data record of the Aman community health workers responsible for door to door visits within assigned catchment area and their status was re-confirmed by the research team staff before enrolling them in the study. The data were collected from February 1, 2016 to June 6, 2016. Written informed consent was obtained from all eligible women who agreed to participate in the study. The ethics review committee of Aga Khan University gave the approval for the study (Fig 1).

For estimating the sample size of the study, the prevalence of various factors (age of women, educational status, and current use of modern method) for controls and cases were ascertained through literature search. In order to determine an odds ratio of at least 2, by taking the lowest proportion of risk factor among controls, i.e. education status (23.9%) [22], with a power of 80%, at a significance level of 5% and with the ratio of 1:2 between cases and controls, a minimum of 125 discontinued users and 249 continued users were required for this study. This sample was increased by 10% to account for the non-responders with a final sample size of 137 cases and 274 controls. The Open Epi software was used to determine the sample size.

Information was collected by using a structured questionnaire which was designed after review of literature and PDHS (2012–2013) questionnaire [21]. The questions were asked in local language ‘Urdu’. The information was gleaned on socio demographic factors, reproductive health, women’s beliefs regarding family planning, and factors related to the use of contraceptives such as access, cost and availability of method of their choice. Data collectors having past experience of data collection with Master or Bachelor’s degree were recruited, who were further trained on research specific needs.

STATA version 12 was used for analysis. Descriptive statistics are presented for both continuous and categorical variables. Chi-square or Fisher exact test was applied for all categorical variables to assess the p-values for differences between cases and controls. For continuous variables, mean with standard deviation (SD) or median with interquartile range were used.

Factor analysis was performed to determine socioeconomic status. Factors with high eigenvalues were considered to classify the socioeconomic status (SES) and SES scores were generated. These were then divided into tertiles. The first tertile (less than -1SD) was considered as lower SES, the last tertile (greater than +1SD) as upper SES and the middle one (greater than -1SD to less than +1SD) as middle SES.

Data were analyzed using logistic regression for both univariate and multivariable analysis. A p-value of 0.25 was considered as criterion for a variable to be significant in univariate analysis. Multi-collinearity between all independent variables was assessed. Logistic regression was used to identify associated factors of contraceptive discontinuation while adjusting for other variables. P-value of less than 0.05 was used for statistical significance. Biologically plausible interactions were assessed with the significance of p value of less than 0.1 as statistically significant for the interaction term. Adjusted odds ratios (ORs) and their 95% confidence intervals (CIs) were used for interpretation of the final results.

## Results

There was no difference in the mean age of the discontinued user (29.3 years SD 5.3) and of the continued user (29.2 years (SD 5.4). A higher proportion (43.8%) of continued users was educated as compared to discontinued users (27.9%). More women in continued users group (48.9%) watched television than women in discontinued user group (43.1%). Employment status did not differ between the two groups (11.7% discontinued user vs 11.6% of the continued user). Regarding ethnicity of the respondents, 36.5% of discontinued users and 48.5% of continued users were Urdu speaking, 19.7% of the discontinued users and 10.4% of the continued users were Sindhi speaking. Moreover, 16.8% discontinued users and 10.5% of the continued users were Pushto speaking.

A greater proportion of husbands of continued user had education of primary and above (75.7%) as compared to husbands of discontinued user (66.4%). Husbands of all the participants were employed at the time of the interview with more of continued user employed as skilled worker than those of discontinued user (21.1% vs 18.8% respectively). Furthermore, 42.3% of discontinued users were living in a joint family system compared to continued users (39.1%). Socioeconomic composite wealth index showed that 27.7% of discontinued user and 18.9% of continued user belonged to low socioeconomic status, while 8.0% of discontinued user and 14.8% of continued user belonged to upper socioeconomic status group. Table 1

**Table 1:** Socio-demographic characteristics of study participants.

Variables	Discontinued user	Continued user
	n = 137	n = 276
	n (%)	n (%)
Age of Woman (years)		
Mean (SD)	29.30± 5.28	29.21± 5.36
Place of residence by Towns		
Korangi	57 (41.61)	142 (51.45)
Bin Qasim	60 (43.80)	97 (35.14)
Landhi	20 (14.60)	37 (13.41)
Educational background of Woman		
No formal education	60 (43.80)	77 (27.90)
Primary or less	14 (10.22)	54 (19.57)
Middle to Secondary	54 (39.42)	103 (37.32)
Intermediate and above	9 (6.57)	42 (15.22)
Reading Newspaper		
Yes	57 (41.61)	126 (45.65)
Not at all	80 (58.39)	150 (54.35)
Frequency of Watching TV		
Daily	59 (43.07)	135 (48.91)
At least once a week	41 (29.93)	81 (29.35)
Occasionally	37 (27.01)	60 (21.74)
Woman’s Employment Status		
Employed	16 (11.68)	32 (11.59)
Not employed	121 (88.32)	244 (88.41)
Ethnicity		
Urdu	50 (36.50)	134 (48.55)
Punjabi	9 (6.57)	19 (6.88)
Sindhi	27 (19.71)	28 (10.14)
Pushto	23 (16.79)	29(10.51)
Hindko	12 (8.76)	31 (11.23)
Others*	16(11.68)	35 (12.68)
Educational background of husband		
No formal education	46 (33.58)	67 (24.28)
Primary	16 (11.68)	34 (12.32)
Middle to Secondary	63 (45.99)	123 (44.57)
Intermediate and above	12 (8.76)	52 (18.84)
Type of Husband’s Employment		
Unskilled	108 (78.83)	203 (73.55)
Skilled	29 (21.17)	73 (26.45)
Family Structure		
Joint Family	58 (42.34)	108 (39.13)
Nuclear family	79 (57.66)	168 (60.87)
SES composite wealth index ṦṦ		
Lower socio-economic status	38 (27.74)	51(18.48)
Middle socio-economic status	88 (64.23)	184 (66.67)
Upper socio-economic status	11 (8.03)	41 (14.86)

* Where others include Balochi, Saraiki, Barmi and Bengali.

**ṦṦ** Socioeconomic Status generated by factor analysis

Table 2 describes the reproductive characteristics of the participants. There were no differences in the reported parity, gravidity and age at the time of first birth between the two groups. However, a larger proportion of women in the continued user group mentioned that their last pregnancy was unplanned as compared to those in the discontinued user group (37.2%vs 29.9% respectively).

**Table 2:** Reproductive characteristics of study participants (February 2016 to June 2016).

Variables	Discontinued user	Continued user
	n = 137	n = 276
	n (%)	n (%)
Duration of current marriage Mean (SD)	10.35±5.82	9.82±5.65
Number of pregnancies (Gravidity)		
Median(IQR)	4 (3,6)	4 (2,5)
Number of live births		
Median(IQR)	3 (2,5)	3 (2,5)
Number of living children		
Median(IQR)	3 (2,4)	3 (2,4)
Age at the time of first birth(years)		
Mean (SD)	20.84±3.33	20.92±3.74
Last pregnancy Unplanned		
Yes	41 (29.93)	103 (37.32)
No	96 (70.07)	173 (62.68)
Information regarding contraceptive given during antenatal visits		
Yes	56 (40.88)	146 (52.90)
No	81 (59.12)	130 (47.10)

Table 3 describes the frequency distribution of last contraceptive method used by the discontinued users and continued users. Mean age at initiating modern contraceptive use was 24.7 years (SD 5.2) for discontinued user group and 23.9 years (SD 4.7) for continued user group. Median number of living children at the time of initiating a modern contraceptive method was similar between both the groups (median 2, IQR: 1, 3 each). The most commonly used last method of contraception by discontinued user was condom (54.7%), injectable (19.7%) IUD and pill (18.9% each) whereas the last most commonly used method by the continued user was also condom (37.7%), IUD (28.3%) and implant (14.1%). A larger proportion of continued user had husband’s support for contraceptive use as compared to the discontinued user (57.6% vs 45.3% respectively). There were no major differences in the place from where both groups obtained the contraceptive method.

**Table 3:** Frequency distribution of last contraceptive use among Discontinued user and Continued user (February 2016 to June 2016).

Variables	Discontinued user	Continued user
	n = 137	n = 276
	n (%)	n (%)
Age at initiating modern contraceptive use (years)		
Mean (SD)	24.75±5.21	23.9±4.70
Number of living children at the time of initiating modern contraceptive use		
Median(IQR)	2 (1,3)	2 (1,3)
Type of the last modern method used		
IUD	26 (18.98)	78 (28.26)
Injectable	27 (19.71)	32 (11.59)
Implant	19 (13.87)	39 (14.13)
Pill	26 (18.98)	23 (8.33)
Condom	39 (28.47)	104 (37.68)
Support for using contraceptive		
Husband and others	62 (45.26)	159 (57.61)
Self-decision	75 (54.74)	117 (42.39)
Source of last method		
Government Source	60 (43.80)	125 (45.29)
Private Source	46 (33.58)	98 (35.51)
Private AMAN	31 (22.63)	53 (19.20)
Counseling by Health care provider/CHW		
Yes	86 (62.77)	170 (61.59)
No	51 (37.23)	106 (38.41)
Information given regarding the side effects		
Yes	68 (49.64)	139 (50.36)
No	69 (50.36)	137 (49.64)
Information given to manage side effects		
Yes	56 (40.88)	126 (45.65)
No	81 (59.12)	150 (54.35)
Ever experienced side effects		
Yes	110 (80.29)	91 (32.97)
No	27 (19.71)	185 (67.03)
Satisfied with the last method		
Yes	28 (20.44)	243 (88.04)
No	109 (79.56)	33 (11.96)
Information given regarding the other methods		
Yes	84 (61.31)	179 (64.86)
No	53 (38.69)	97 (35.14)

Almost an equal proportion of women in both the groups were counseled for family planning FP methods by the health care provider (discontinued user 62.8% vs continued user 61.6%, and were informed about the side effects of the methods (49.6% in discontinued user group vs 50.4% in continued user group). However, for the last method used, a larger proportion of women in discontinued user group reported side effects as compared to the women in the continued user group (80.3% as compared to 33.0% respectively). A higher proportion of continued user was satisfied by the last method they used (88.0%) as compared to the discontinued user (20.4%). Not all women in both the groups were counseled for all family planning methods, however, for those who were counseled, about 65% women were in the continued user group as compared to discontinued group (61%).

Table 4 describes the access to contraceptive methods. Nearly, 37% of women in continued user group mentioned source of modern method of contraception far from their home as compared to less than a third (32.1%) of discontinued users. Nearly 14% of discontinued users reported that they faced difficulty in accessing contraceptive by themselves as compared to 25.4% of continued user. Nearly all the women in both the groups mentioned treatment of side effects as costly at private clinic (89.1% discontinued user vs 86.6% continued user) whereas only 5.1% of discontinued user and 3.6% of continued user considered treatment at government facilities costly. Also 70.8% of discontinued user and 37.7% of continued user considered travelling to reach clinics for treatment of complications difficult.

**Table 4:** Factors related to access and cost of contraceptive use.

Variables	Discontinued user	Continued user
	n = 137	n = 276
	n (%)	n (%)
Long distance to access contraceptives		
Yes	44 (32.12)	104 (37.68)
No	93 (67.88)	172 (62.32)
Difficulty in accessing contraceptives by themselves		
Yes	19 (13.87)	70 (25.36)
No	118 (86.13)	206 (74.64)
Consultation/treatment for FP use at Private clinics considered to be costly		
Yes	122 (89.05)	239 (86.59)
No	15 (10.95)	37 (13.41)
Consultation/treatment for FP use at Government clinics considered to be costly		
Yes	7 (5.11)	10 (3.62)
No	130 (94.89)	266 (96.38)
Difficulty in traveling to reach clinics for treatment of complications		
Yes	97 (70.80)	104 (37.68)
No	40 (29.20)	172 (62.32)

Table 5 shows the factors associated with discontinuation of modern contraceptive method use. On univariate analysis, women’s level of education, belonging to Sindhi and Pushto ethnic groups, husband’s level of education, lack of husband’s support, low SES, receiving no counseling of FP during antenatal care, using IUD, experiencing side effects, difficulty in traveling to reach clinics and difficulty in accessing contraceptive by themselves were associated with discontinuation of the method (p value less than 0.25) and were included in multivariable model.

**Table 5:** Multivariable analysis for the association of different factors by discontinuation status.

Variables	Unadjusted OR	95% CIs	Adjusted OR	95% CIs
Ethnicity				
Punjabi	1.26	0.53–2.99	0.72	0.26–1.98
Sindhi*	2.58	1.38–4.80	2.54	1.16–5.57
Pushto	2.12	1.12–4.01	2.13	0.94–4.80
Hindko	1.03	0.49–2.17	0.97	0.37–2.50
Others	1.22	0.62–2.40	1.41	0.61–3.27
Urdu	1 (Reference)		1 (Reference)	
Type of the modern method last used				
IUD*	0.88	0.49–1.58	0.20	0.08–0.49
Injectable	2.25	1.19–4.22	0.66	0.26–1.67
Implant*	1.29	0.67–2.51	0.27	0.10–0.71
Pill	3.01	1.54–5.89	1.49	0.62–3.60
Condom	1 (Reference)		1 (Reference)	
Support for using contraceptive				
Husband*	0.60	0.40–0.91	0.58	0.34–0.98
Self-decision	1		1	
Ever experienced side effects				
Yes*	8.28	5.07–13.52	15.12	7.50–30.51
No	1 (Reference)		1 (Reference)	
Difficulty in accessing contraceptives by themselves				
Yes*	0.47	0.27–0.82	0.40	0.19–0.83
No	1 (Reference)		1 (Reference)	
Difficulty in traveling to reach clinics for treatment of complications				
Yes*	4.01	2.57–6.23	4.10	2.38–7.05
No	1 (Reference)		1 (Reference)	

* Variables are statistically significant

On multivariable analysis after adjusting for all other variables in the model, the factors which were independently associated with discontinuation of a modern method of contraception were belonging to Sindhi ethnic group [OR: 2.54; 95% CI (1.16–5.57], support from husband for use of contraceptive [OR: 0.58; 95% CI 0.34–0.98], experiencing side effects [OR: 15.12; 95% CI 7.50–30.51], difficulty in accessing contraceptive by themselves [OR: 0.40; 95% CI 0.19–0.83] and having difficulty in traveling to reach clinics for management of side effects [OR: 4.10; 95% CI 2.38–7.05].

## Discussion

The study results identified a discontinued user as ‘a Sindhi speaking woman; one who had experienced side effects of the last method used; one who was not supported by her husband for using contraception and one who found it difficult to travel by themselves to reach clinics for the management of side effects.

Experiencing side effects of a method has been well documented in the literature as a major cause of discontinuing a modern method of contraception [16]. More than 80% of the discontinued users in this study reported experiencing side effects of the last method they used before discontinuing. The side effects were mostly related to IUDs, implants, pills and injectable. We believe that if the women are not counseled appropriately about the side effects at the time they have selected the method and not communicated what to expect as a side effect and what remedies to adopt while experiencing anything unusual related to contraceptive intake or when to consult back to health care provider then there is a high probability that such a method will be discontinued soon [6,12]. The main side effects experienced by women were reported by various studies are irregular bleeding, headache, absence of menstruation, weight gain and depression resulting in discontinuation of the selected method [23]. The discontinuation rate due to side effects vary from 2% in Armenia to 37% in Egypt [7] and in some countries has been identified as a public health issue as this leads to unplanned, unwanted pregnancy which might lead to termination of pregnancy by unsafe means [5–6,24].

In this study sample Sindhi speaking women were more prone to discontinuation of use of modern method of contraception as compared to other groups. Although in our study Pashtuns were two times more likely to be discontinued user but findings were not significant. To the best of our knowledge, studies conducted in Pakistan have not highlighted ethnic differences in contraceptive discontinuation. Findings from international studies were inconsistent with this study findings showing that racial or ethnic differences do not have any role in discontinuation of a method [25– 26]. This study results differ, may be due to the fact that in the area where study was conducted, Sindhi speaking population was in minority as compared to other ethnic groups (11.1%) [22] or alternatively this could be the reality and ethnic differences for discontinuation needs to be explored further.

One of the important but often overlooked component of fertility management is the role which husbands play in family planning decision making [27]. Literature suggests significant association between Couples’ role on decision making to use modern contraceptive method and its continuation [23]. This study revealed that women supported by their husband are less likely to discontinue. In addition, discontinuation was observed less among the women who used long-acting reversible contraceptive (LARC) methods as compared to short-acting methods. In Ethiopia, the highest discontinuation rate was seen for the injectable (67.2%), and pills (27.4%) [23]. Similarly, findings from the study conducted in Nairobi reported the condom and pill users had highest discontinuation rates, while implants were least discontinued [26]. Present study findings do not show such pattern because this is a case control study and both groups were selected with some definitive selection criteria.

In this study sample, difficulty in traveling to reach clinics for treatment of complications was strongly associated with contraceptive discontinuation. Frequent travelling adds to the cost of using a method and poor populations would tend to drop using a method than to continue seeking care. Present study suggests that those women who had difficulty in accessing contraceptive by themselves were less likely to discontinue. These women in study were mostly using long acting contraceptives, which not only were providing them with long duration of contraception but were also saving them from repeated visits.

In conclusion side effects of a modern method and the management of its side effects are the major reasons for discontinuation of a modern method of contraception in Sukh Initiative populations. Study findings emphasize on the appropriate trainings, counseling of FP service providers and referrals for quality of care for family planning services. Trainings on appropriate management of the side effects remain the most neglected area of family planning service, which in turn results in ever increasing proportion of ever users of family planning methods and an increasing gap in the current and ever use of family planning methods. Moreover, we recommend that male involvement in family planning and their support in use of contraceptive method played important role in continuation of contraceptive use so couple counseling should be promoted in order to reduce discontinuation.

## Supporting information

S1 File Questionnaire in Urdu. (DOC)

S2 File Questionnaire in English. (DOC)

S3 File Data for Journal. (ZIP)
