# Supplementary material for: Factors associated with the discontinuation of modern methods of contraception in the low income areas of Sukh Initiative Karachi: A community-based case control study
Source: PLoS One. 2019 Jul 3;14(7):e0218952. doi: 10.1371/journal.pone.0218952 (PMC6608957; doi:10.1371/journal.pone.0218952)
Supplement: S1 File — (DOC) [file pone.0218952.s001.doc]

**کراچی پاکستان کے کم آمدنی والے علاقے میں افزائش نسل گروپ سے تعلق رکھنے والی خواتین کے حمل کو روکنے کے جدید طریقوں کو ترک کرنے سے متعلق عناصر**

***سوالنامہ***

***آغا خان یونیورسٹی ، ہسپتال***

| ***شادی شدہ خواتین کا سوالنامہ*** | | | | |  |
| --- | --- | --- | --- | --- | --- |
| **لانڈھی بن قاسم ملیر کورنگی** | | | ٹاؤن کا نام | |  |
|  | | | **ایریا کا نام** | |  |
|  | | | گھرانہ نمبر | |  |
|  | | | **عورت کا نام** | |  |
|  | | | **شوہر کا نام** | |  |
|  | | | **فون نمبر** | |  |
|  | | | انٹر ویور کا نام | |  |
| دن ­­______ مہینہ2015________ | | | **انٹرویو کی تاریخ** | |  |
|  | | | **سپروائزر کا نام** | |  |
| 2015 ­­______ مہینہ________ دن | | | **فیلڈ ڈیٹا ایڈ یٹنگ کی تاریخ** | |  |
|  | | | **ڈیسک ایڈیٹر** | |  |
|  | | | **ڈیٹا انٹری کی تاریخ** | |  |
|  | | | **انٹرویو کا نتیجہ**  1. مکمل  2. نا مکمل  3. گھر پر نہیں  4. انکار کردیا  5. گھر نہیں ملا | |  |
| **جوابات** | **Section: B Respondent’s Background Characteristics** | | |  | |
|  | | | | | |
|  |  | آپ کی عمر کتنی ہے ؟(مکمل سالوں میں) | | **RB1** | |
|  | 1. شوہر ساتھ رہتے ہیں  2. کہیں اور رہتے ہیں | کیا آپ کے شوہر آپ کے ساتھ رہتے ہیں یا کہیں اور؟ | | **RB2** | |
|  |  | موجودہ شادی کی مّدت  (مکمل سالوں میں) | | **RB3** | |
|  |  | آپ کی پہلے شادی کے وقت آپ کی عمر کیا تھی؟ (مکمل سالوں میں) | | **RB4** | |
|  | 1. ہاں  2. نہیں | کیا آپ کبھی اسکول گئیں ہیں؟ | | **RB5** | |
|  |  | آپ نے تعلیم کس درجے تک حاصل کی ؟ | | **RB6** | |
|  | 1. روزانہ  2. ہفتے میں ایک بار  3. کبھی کبھار  4. کبھی بھی نہیں | کیا آپ اخبار پڑھتی ہیں ؟  (روزانہ ، کم از کم ہفتے میں ایک بار ، کبھی کبھار ، کبھی بھی نہیں ) | | **RB7** | |
|  | 1. روزانہ  2. ہفتے میں ایک بار  3. کبھی کبھار  4. کبھی بھی نہیں | کیا آپ ریڈیو سنتی ہیں؟  (روزانہ ، کم از کم ہفتے میں ایک بار ، کبھی کبھار ، کبھی بھی نہیں ) | | **RB8** | |
|  | 1. روزانہ  2. ہفتے میں ایک بار  3. کبھی کبھار  4. کبھی بھی نہیں | کیا آپ ٹی وی دیکھتی ہیں ؟  (روزانہ ، کم از کم ہفتے میں ایک بار ، کبھی کبھار ، کبھی بھی نہیں) | | **RB9** | |
| **پر جائیں RB13 اگر ہاں تو** | 1. ہاں  2. نہیں | کیا آپ کے پاس ذاتی موبائل فون ہے ؟ | | **RB10** | |
| **پر جائیں** **RB14 اگر نہیں تو** | 1. ہاں  2. نہیں | اگر آپ کے پاس موبائل فون نہیں تو کیا آپ کسی اور کا موبائل فون استعمال کرتی ہیں ؟ | | **RB11** | |
|  | 1. شوہر کا  2. پڑوسی کا  3. دوست کا  ـــــــــــــــــــــــــــــــــــ 96. کوئی اور | اگر ہاں تو کس کا موبائل فون استعمال کرتی ہیں عام طور پر ؟ | | **RB12** | |

|  | 1. روزانہ  2. ہفتے میں ایک بار  3. کبھی کبھار  4. کبھی بھی نہیں | کیا آپ موبائل فون استعمال کرتی ہیں روزانہ /کم سے کم ہفتے میں ایک بار / کبھی کبھار / بالکل بھی نہیں ؟ | **RB13** |
| --- | --- | --- | --- |
|  | اردو 1.  2. پنجابی  3. سندھی  4. پشتو  5. بلوچی  6. انگلش  7. بروہی  8. سرائیکی  9. ہند کو  10.کشمیری  11. پہاڑی  12. پوٹو ہاری  13. مار واڑی  14. فارسی  96. دیگر ، وضاحت ـــــــــــــــــــــ | آپ کی مادری زبان کیا ہے؟ | **RB14** |

| **جوابات** | **Section: C Socio-demographic Information** | |  |
| --- | --- | --- | --- |
|  | | | |
|  | 1. ذاتی مکان  2. کرائے کا  3. دیگر ،وضاحت کریں ــــــــــــــــــــ | آپ جس مکا ن میں رہتی ہیں اس کی مالکا نہ حیثیت کیا ہے ؟ | **SD1** |
|  |  | آپ کے گھر میں باورچی خانے / لیٹرین اور گیراج کے علاوہ کتنے کمرے ہیں؟ | **SD2** |
|  | 1. ہاں  2. نہیں  96. دیگر ، وضاحت ـــــــــــــــــــــ | کیا آپ آج کل آمدنی حاصل کرنے کے لئے کام کررہی ہیں؟ | **SD3** |
|  | 1. کھیتوں کا کام  2. استانی  3. گھروں میں کام کرتی ہوں  4. لیڈی ہیلتھ ورکر/نرس/ہیلتھ کئیر ورکر  96. دیگر ، وضاحت ـــــــــــــــــــــ | آپ کا کام کس قسم کا ہے؟ | **SD4** |
|  | 1. ہاں  2. نہیں | آپ شادی سے پہلے آمدنی حاصل کرنے کے لئے کام کر رہی تھی؟ | **SD5** |
|  | 1. آپ خود  2. شوہر  3. ساس  4. سسر  96. دیگر ، وضاحت ـــــــــــــــــــــ | جب آپ بیمار ہوتی ہیں تو آپ کا علاج کرنے کا فیصلہ کرن کرتا ہے؟ | **SD6** |
|  | 1. ہاں  2. نہیں | کیا آپ کے شوہر پیسے کمانے کے لئے کام کرتے ہیں؟ | **SD7** |
|  | 1. کسان  2. مچھیرا  3. استاد  4. ہوٹل میں کام کرنے والا  5. مزدور  6. دُکاندار  96. دیگر ، وضاحت ـــــــــــــــــــــ | اگر ہاں تو اُن کا پیشہ کیا ہے؟ | **SD8** |
|  | روپو میں | آپ کے گھر کی مجموعی آمدنی کتنی ہے؟ | **SD9** |

|  | 1. بجلی  2. ریڈیو  3. ٹیلی ویژن  4. ٹیلیفون  5. ریفر یجریٹر  6. الماری  7. کرسی  8. روم کولر  9. ائیر کنڈیشن  10. واشنگ مشین  11. واٹر پمپ  12. بیڈ  13. گھڑیال  14. صوفہ  15. کیمرہ  16. سلائی مشین  17. کمپیوٹر  18. انٹرنیٹ  19. کوئی بھی نہیں | اب میں آپ سے گھریلو اشیا کے بارے میں سوال کروں گی ان میں سے جو اشیا موجود ہوں آپ کے گھر میں ان کے بارے میں بتائیں؟  **(جو چیز موجود ہے اس پر دائرہ بنائیں )** | **SD10** |
| --- | --- | --- | --- |
| اگر ہاں ،تو نمبر لکھیں | 1. گھڑی  2. موبائل فون  3. سائیکل  4. اسکوٹر  5. ٹرک / بس / ریڑھی  6. ٹریکٹر  7. کشتی موٹر والی  8. کشتی بغیر موٹر والی  9. کوئی بھی نہیں | کیا آپ کے گھر کے کسی بھی فرد کے پاس ان ذاتی اشیا میں سے کچھ موجود ہے ؟  (جو اشیا موجود ہے اس پر د ائرہ بنائیں) | **SD11** |
| اگر ہاں ، توکتنے ایکڑ (اصل ایکڑ کی تعداد لکھیں) | 1. ہاں  2. نہیں | کیا آپ کے گھر کا کوئی فرد زرعی زمین کا مالک ہے؟ | **SD12** |
|  | 1. گائے  2. بھینس  3. بکریاں  4. بیل  5. بھیڑ  6. اونٹ  7. مرغیاں  مندرجہ بالا میں سے کوئی بھی جانور نہیں | آپ ان میں سے کسی مویشی کی ملکیت رکھتے ہیں؟ | **SD13** |

| **جوابات** | **Section: D Reproductive Health** | |  |
| --- | --- | --- | --- |
|  | | | |
| **RH18** اگر نہیں تو  پر جائیں | 1. ہاں  2. نہیں | کیا آپ نے کبھی کسی بچے کو جنم دیا ہے ؟ | **RH1** |
|  | 1. ہاں  2. نہیں | کیا آپ کے یہاں پیدا ہونے والے بیٹے یا بیٹیوں میں سے کوئی اس وقت آپ کے ساتھ رہتا ہے ؟ | **RH2** |
|  |  | زندہ بچوں کی تعداد کتنی ہے ؟ | **RH3** |
|  | 1.لڑکے جو ساتھ ہیں  2. لڑکیا ں جو ساتھ ہیں | کتنے بیٹے آپ کے ساتھ رہتے ہیں ؟  کتنی بیٹیاں آپ کے ساتھ رہتی ہیں ؟  **(اگر نہیں تو 00 ریکارڈ کریں )** | **RH4** |
| **RH7** اگر نہیں تو  پر جائیں | 1. ہاں  2. نہیں | کیا آپ کا کوئی بیٹا یا بیٹی ہے جسے آپ نے جنم دیا ہو لیکن آپ کے ساتھ نہ رہتا ہو ؟ | **RH5** |
|  | 1. بیٹوں کی تعداد  2. بیٹیوں کی تعداد | کتنے بیٹے ایسے ہیں جو زند ہ ہیں لیکن ساتھ نہیں رہتے ؟  کتنی بیٹیاں ایسی ہیں جو زند ہ ہیں لیکن ساتھ نہیں رہتی ؟  **(اگر نہیں تو 00 ریکارڈ کریں )** | **RH6** |
| **RH9** اگر نہیں تو  پر جائیں | 1. ہاں  2. نہیں | کیا آپ کے یہاں کسی ایسے بچے کی پیدائش ہوئی ہے جو زندہ پیدا ہو ہو اور بعد میں فوت ہوگیا ہو ؟ | **RH7** |
|  | بیٹے  بیٹیاں | کتنے بیٹے فوت ہوچکے ہیں ؟  کتنی بیٹیاں فوت ہوچکی ہیں ؟  **اگر نہیں تو 00 ریکارڈ کریں** | **RH8** |
| **RH11** اگر نہیں تو  پر جائیں | 1. ہاں  2. نہیں | کیا کبھی آپ کے حمل کے نتیجے میں ایسا ہوا کہ حمل ضائع ہوگیا ہو زندہ بچے کی پیدائش نہ ہوئی ہو یا مردہ بچہ پیدا ہوا ہو ؟ | **RH9** |
|  |  | آپ کے کتنے حمل ایسے تھے جن کے نتنجے میں زندہ بچے کی پیدائش نہیں ہوئی ہو یا حمل ضائع ہوگئے ہوں ؟ | **RH10** |
|  |  | ایک حاملہ عورت کو اپنے حمل کے دوران کتنی بار معائنہ کروانا چاہئے ؟ | **RH11** |
|  |  | آپ نے آخری بچے کی پیدائش کے وقت حمل کے دوران کتنی بار معائنہ کروایا؟ | **RH12** |
| اب میں آپ سے اب تک کے تمام حمل اور زندہ یا مردہ بچوں کے بارے میں دریافت کروں گی ۔ | | | **RH13** |

**
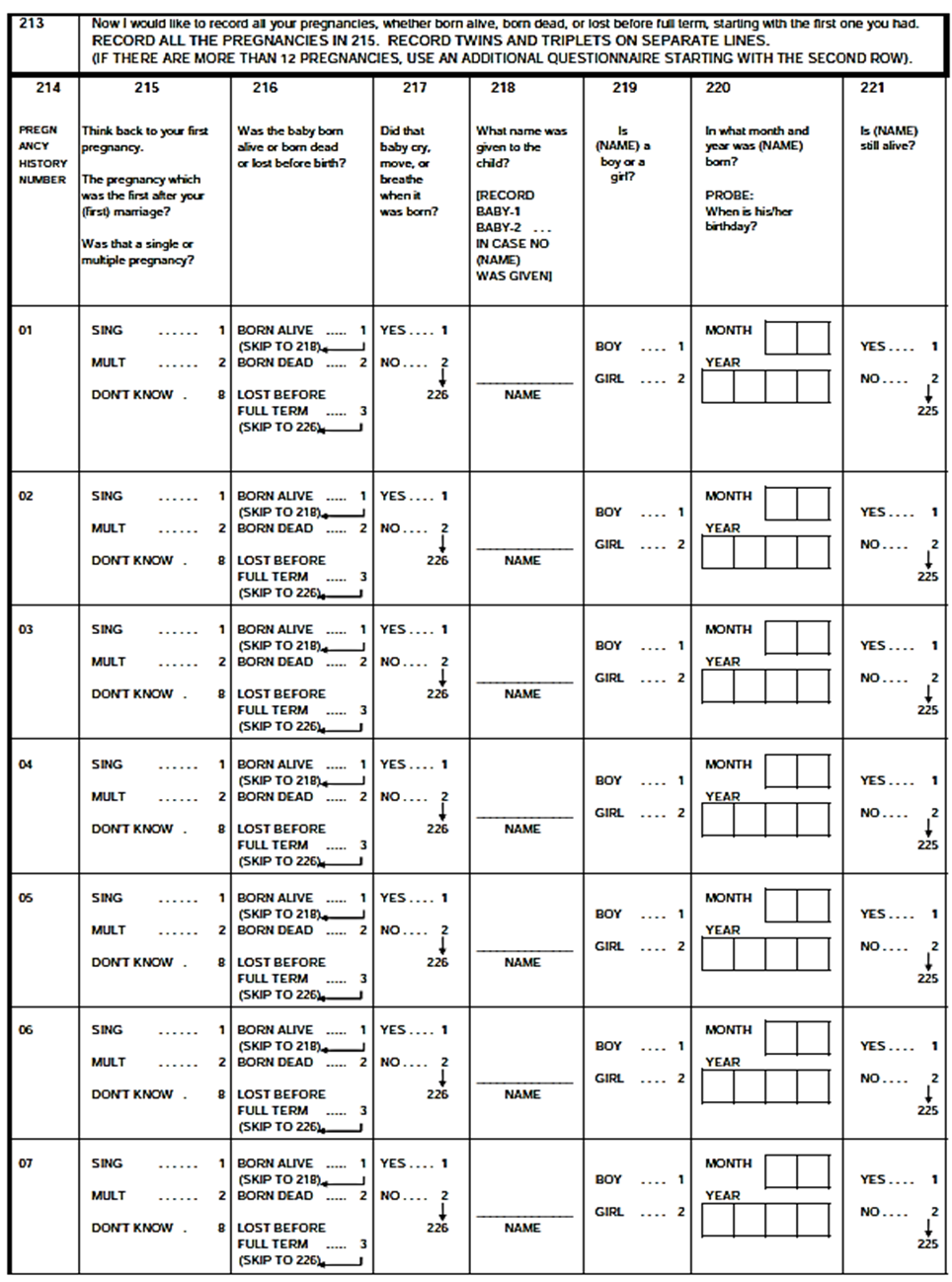
**

**
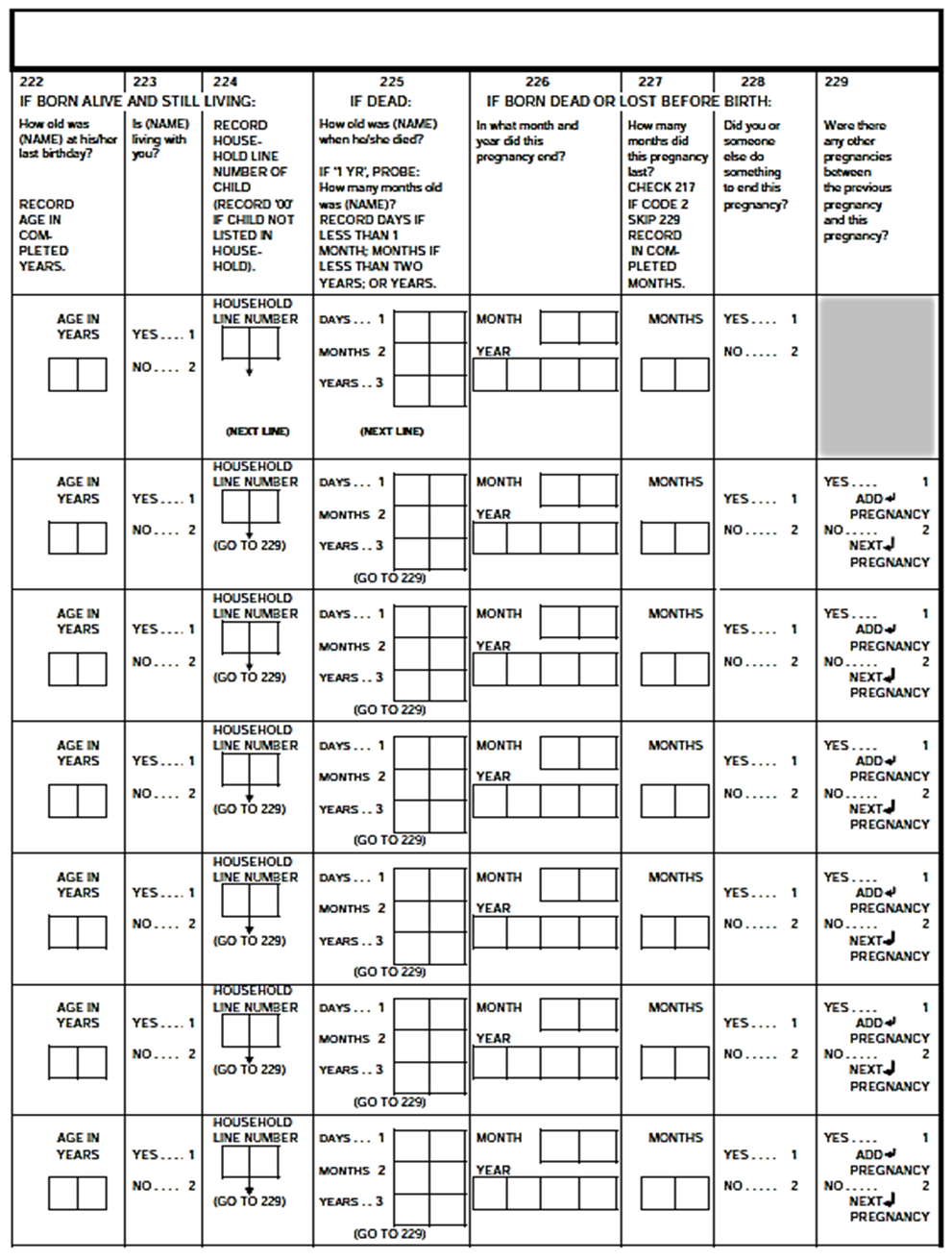
**

|  |  | پہلے بچے کی پیدائش کے وقت آپ کی عمر کیا تھی ؟ | **RH14** |
| --- | --- | --- | --- |
| **RH17** اگر ہاں تو  پر جائیں | 1. ہاں  2. نہیں | حب آپ حاملہ ہوئیں تو کیا اس وقت حاملہ ہونا چاہتی تھیں ؟ | **RH15** |
|  | 1. کچھ عرصے بعد  2. مزید بچوں کی خواہش نہیں | کیا آپ کچھ عرصے کے بعد بچہ چاہتی تھی یا آپ کو بچے کی خواہش نہیں تھی ؟ | **RH16** |
|  |  | آپ کے سب سے چھوٹے بچے کی عمر کتنی ہے ؟ | **RH17** |
|  |  | آپ کے خیال میں حمل کے وقت عورت کی عمر کتنی ہونی چاہئے ؟ | **RH18** |
|  |  | کیا آپ جانتی ہیں کہ کس عمر کے بعد عورت کو مزید حاملہ نہیں ہونا چاہئے ؟ | **RH19** |
|  | 1. کم از کم 24 مہینے  2. 36 مہینے  3. زیادہ سے زیادہ 5 سال  98. نہیں معلوم  96. دیگر ، وضاحت ـــــــــــــــــــــ | ماں اور بچے کی اچھی صحت کے لئے بچے کی پیدائش کے بعد اگلے حمل کےلئے کتنا وقفہ ضروری ہے؟ | **RH20** |
|  | 1. حمل کی پیچید گیوں میں کمی  2. اگلے حمل کے لئے ما ں کو تیار کرنا  3. صحت مند بچے کی پیدائش کے امکانات بڑھ جاتے ہیں  4. بچے کو دودھ پلانے کے لئے مناسب وقت وقت ملتا ہے  5. چھاتی کے سرطان کے خطرے کو کم کرتا ہے  96. اور کوئی  98. نہیں معلوم  99. یاد نہیں | بچوں کی پیدائش میں وقفے سے ماں کو کونسے فائدے حاصل ہوسکتے ہیں؟ | **RH21** |
|  | 1. نوزائیدہ بچے کی صحت اچھی ہوگی  2. نوزائیدہ بچے کی بستر دیکھ بھال  3. مناسب مدت تک ماں کا دودھ پی سکتا ہے  4. ماں کی زیادہ توجہ ملتی ہے  5. ماں اور بچے کے دوران پیار بڑھتا ہے  96. اور کوئی  98. نہیں معلوم  99. یاد نہیں | **بچوں کی پیدائش میں وقفے سے نوزائیدہ بچے کو کیا فائدے حاصل ہوسکتے ہیں ؟** | **RH22** |
|  | 1. خاندان کی فلا ح وبہبود  2. شوہر اور بیوی کو اطمینان ملتا ہے خاندانی خوشحالی کے ذریعے  3. خاندان کو معاشی ترقی کا موقع ملتا ہے  96. اور کوئی  98. نہیں معلوم  99. یاد نہیں | بچوں کی پیدائش میں وقفے سے خاندان کو کیا فائدے حاصل ہوتے ہیں؟ | **RH23** |

| **جوابات** | | **Section: E Contraceptive Use** | |  |
| --- | --- | --- | --- | --- |
|  | | | | |
| **CU1A.**  کیا آپ نے کبھی کوئی طریقہ استعمال کیا ؟ | | اب میں آپ سے خاندانی منصوبہ بندی کے بارے میں اور خاندانی منصوبہ بندی کے طریقوں کے بارے میں سوالات کروں گی۔ | | **CU1** |
| کیا آپ نے کبھی (نل بندی) بچوں کی پیدائش کو روکنے کے لئے آپریشن کروایا؟  1. ہاں  2. نہیں | | 1. ہاں (اگر نہیں تو استعمال کے طریقے کو چھوڑ دیں پوچھنا)  2. نہیں | نل بندی : خواتین ایک اپریشن کے ذریعے بچے کی پیدائش کو مستقل طور پر روک سکتی ہے۔ | **CU1.1** |
| کیا آپکے شوہر نے کبھی نس بندی کا آپریشن کروایا ؟  1. ہاں  2. نہیں | | 1. ہاں  2. نہیں | نس بندی : مرد ایک آپریشن کے ذریعے بچوں کی پیدائش کو مستقل طور پر روک سکتے ہیں۔ | **CU1.2** |
| 1. ہاں  2. نہیں | | 1. ہاں  2. نہیں | بچوں میں وقفے کے لئے کسی ماہر ڈاکٹر / ہیلتھ ورکر سے خواتین ایک تار / چھلہ / رمگ اندر رکھواسکتی ہیں جو حمل کو لمبے عرصے تک روک سکتا ہے ؟ | **CU1.3** |
| 1. ہاں  2. نہیں | | 1. ہاں  2. نہیں | ٹیکے : خواتین بچوں میں وقفے کے لئے کسی ڈاکٹر یا ہیلتھ ورکر سے ٹیکا لگواسکتی ہیں جو حمل کو ایک یا زیادہ مہینوں کے لئے روک سکتا ہے ؟ | **CU1.4** |
| 1. ہاں  2. نہیں | | 1. ہاں  2. نہیں | کیپسول / امپلانٹ : خواتین بچوں میں وقفے کے لئے کسی ڈاکٹر یا ہیلتھ ورکر کی مدد سے بازو میں چھوٹے چھوٹے کیپسول لگواتی ہیں جو حمل کو لمبے عرصے تک روک سکتا ہے ؟ | **CU1.5** |
| 1. ہاں  2. نہیں | | 1. ہاں  2. نہیں | گو لی: خواتین روزانہ ایک ٹیبلیٹ / گولی کھا سکتی ہیں جس کا مسلسل استعمال بچوں میں وقفہ کے لئے مئو ثر ہے ؟ | **CU1.6** |
| 1. ہاں  2. نہیں | | 1. ہاں  2. نہیں | شوہر سے تعلق کے بعد عورت بچوں کی پیدائش میں وقفے اور حمل کو روکنے کے لئے تین دن کے اندر ایک مانع حمل گولی کھا سکتی ہے (یہ طر یقہ صرف ہنگامی صورت حال کے لئے ہے ) | **CU1.7** |
| 1. ہاں  2. نہیں | | 1. ہاں  2. نہیں | ربڑ / کونڈم : مرد بیوی سے تعلق کے وقت اپنے خاص حصے پر ایک ربڑ پہن سکتا ہے جو کہ بچوں کی پیدائش میں وقفے کے لئے مئو ثر ہے ؟ | **CU1.8** |
| 1. ہاں  2. نہیں | | 1. ہاں  2. نہیں | بچوں کی پیدائش میں وقفے کے لئے عورت ایک رنگین دانوں والے تار کی مدد سے اپنے ان خاص دنوں کا حساب رکھتی ہے جن میں وہ حاملہ ہوسکتی ہے ان خاص دنوں میں میاں / بیوی احتیاط کرتے ہیں ربڑ کے ذریعے یا پھر دور رہ کر۔ | **CU11.9** |
| 1. ہاں  2. نہیں | | 1. ہاں  2. نہیں | عورت اپنے بچوں کو اپنا دودھ پلا کر اگلے حمل میں وقفہ کرسکتی ہے۔ | **CU1.10** |
| 1. ہاں  2. نہیں | | 1. ہاں  2. نہیں | عورت ہر مہینے ماہواری کے دن کا حساب رکھ کر ان خاص دنوں میں احتیاط کرسکتی ہے جب حاملہ ہوسکتی ہے۔ | **CU1.11** |
| 1. ہاں  2. نہیں | | 1. ہاں  2. نہیں | مر د بچوں کی پیدائش مین وقفے کے لئے احتیاط کرسکتے ہیں اگر وہ اپنا پانی اندر نہ چھوڑیں۔ | **CU1.12** |
|  | | | | |
| **CU16** اگر نہیں تو  پر جائیں | | 1. ہاں  2. نہیں | کیا آپ نے ابھی بتائے گئے طریقوں میں سے کسی بھی طریقے کو استعمال کیا ہے؟ | **CU2** |
|  | | ______________ آخری طریقہ کانام | آپ نے آخری طریقہ کون سا استعمال کیا؟ | **CU3** |
|  | | شروع کرنے کا سال  شروع کرنے کا میینہ  مکمل دورانیہ | کونسے سال سے آپ نے وہ طریقہ استعمال کرنا شروع کیا کتنے سالوں تک وہ طریقہ استعمال کیا (مکمل سالوںمیں) | **CU3A** |
|  | | 1. نل بندی  2. آئی یو ڈی  3. انجیکشن  4. بازو میں رکھنے والا کیپسول  5. روزانہ والی گولی  6. کنڈم  7. رنگین دانوں والے تار کی مدد سے دنوں کا حساب رکھنا  8. بچے کو اپنا دودھ پلا کر اگلے حمل کے لئے وقفہ  9. عورت ہر مہینے ماہواری کے دن کا حساب رکھ کر احتیاط کرلے  10. انزال / عُزل  96. دیگر ، وضاحت | آخری دو طریقے بتائیں اگر مختلف طریقے استعمال کئے ہے تو؟س | **CU3B** |
|  | |  | آپ نے جب پہلے دفعہ خاندانی منصوبہ بندی کے طریقہ کا استعمال شروع کیا اُس وقت آپ کی عمر کیا تھی؟(مکمل سالوں میں) | **CU4** |
|  | |  | جب آپ نے پہلی دفعہ خاندانی منصوبہ بندی کا طریقہ استعمال کیا اُس وقت آپ کے زندہ بچے کتنے تھے؟ | **CU5** |
| **CU6B** اگر نہیں تو  پر جائیں | | 1. ہاں  2. نہیں | کیا آپ اس وقت حمل میں تاخیر یا حمل کو روکنے کے لئے خاندانی منصوبہ بندی کا آخری طر یقہ استعمال کررہی ہیں؟ | **CU6** |
| پر جائیں **CU7** | | 1. چھ ماہ سے زیاہ  2. چھ ماہ | اگر ہاں تو کتنے مہینوں سے آپ خاندانی منصوبہ بندی کا آخری طر یقہ استعمال کررہی ہیں(بغیر چھوڑیں ہوئے) | **CU6A** |
|  | | 1. چھ ماہ سے زیاہ  2. چھ ماہ | اگر نہیں تو کتنے مہینو ں سے آپ خاندانی منصوبہ بندی کا کوئی بھی طریقہ استعمال نہیں کررہی؟ | **CU6B** |
|  | | 1. بنیادی طور پر میرا  2. بنیادی طور پر شوہر کا  3. مل کے فیصلہ  96. دیگر وضاحت کریں | خاندانی منصوبہ بندی کے طریقے کے استعمال کا فیصلہ آپ کا اپنا ہے یا آپ کے شوہر کا فیصلہ ہے یا آپ دونوں کا؟ | **CU7** |
|  | | 1. گورنمنٹ / آر ایچ ایس اے  2. رورل ہیلتھ سینٹر  3. فیملی ویلفئیر سینٹر / ایم سی ایچ  4. ڈسپنسری / فارمیسی  5. موبائل سروس کیمپ / یونٹ  6. لیڈی ہیلتھ ورکر  7. لیڈی ہیلتھ وزیٹر  8. بیسک ہیلتھ یونٹ  9. میل موبلا ئزر  10. فیملی ویلفئر اسسٹنٹ  11. کمیونٹی ہیلتھ ورکر  96. دیگر ، وضاحت | جب آپ نے آخری طریقہ استعمال کرنا شروع کیا تو آخری طریقہ اُس وقت کہاں سے حاصل کیا؟ | **CU8** |
|  | | 1. ہاں  2. نہیں | کیا اس وقت آپکو اس (زیر استعمال ) طریقے کے ممکنہ مضر اثرات کے بارے میں بتایا گیا تھا ؟ | **CU9** |
|  | | 1. ہاں  2. نہیں | کیا آپکو بتایا گیا تھا کہ موجودہ طریقے کے استعمال ہونے والے مضر اثرات کی صورت میں آپ کو کیا کرنا ہوگا۔ | **CU10** |
|  | | 1. ہاں  2. نہیں | کیا کبھی آپکو موجودہ طریقے کے استعمال کے نتیجے میں کسی بھی قسم کے مضر اثرات کا سامن کرنا پڑا ؟ | **CU11** |
|  | | 1. سر درد  2. متلی / چکر  3. خون کا زیادہ پہنا  4. خون کے دھبے  6. ماہواری کا خراب ہونا  7. ذہنی دباؤ  96. دیگر ، وضاحت  99. یاد نہیں | **آپ کو کس طرح کے مضر اثرات کا تجربہ ہوا ؟** | **CU12** |
| **CU15** اگر نہیں تو  پر جائیں | | 1. ہاں  2. نہیں | کیا آپ نے مضراثرات کی صورت میں کسی بھی قسم کا علاج / طبی مشورہ حاصل کیا؟ | **CU13** |
|  | | **پبلک سیکٹر**  1. گورنمنٹ / آر ایچ ایس اے  2. رورل ہیلتھ سینٹر  3. فیملی ویلفئیر سینٹر  4. ایم سی ایچ  5. ڈسپینسری  6. موبائل سروس کیمپ  7. لیڈی ہیلتھ ورکر  8. لیڈی ہیلتھ وزیٹر  9. بیسک ہیلتھ یونٹ  10. میل موبلا ئزر  11. ایف ڈبلیو اے  96. دیگر ، پبلکــــــــــــــــــــــــــــــ  **پرائیوٹ /این جی او میڈیکل سیکٹر**  12. فارمیسی ، کیمسٹ  13. پرائیوٹ ڈاکٹر  14. ہومیو پیتھک  15. ڈسپینسر / کمپائو نڈر  96. دیگر پرائیوٹ میڈیکل ـــــــــــــــــ  16. دکان (فارمیسی اور ڈسپیسری کے علاوہ)  17. دوست / رشتہ دار  18. حکیم  19. دائی ، ٹریڈ برتھ اٹینڈنٹ  96. دیگر وضاحت ــــــــــــــــــــــــ  98. نہیں معلوم  99. یاد نہیں | آپ نے طّبی مشورہ / علاج کہاں سے حاصل کیا ؟ | **CU14** |
|  | | 1. ضروری نہیں  2. بہت مہنگا تھا  3. بہت دوور ہے  4. ٹرانسپورٹ نہیں تھی  5. کوئی ساتھ لے جانے کے لئے نہیں تھا  6. سہولت کا معیار اچھا نہیں  7. وقت نہیں تھا  8. معلوم نہیں کہا ں جائیں  9. لیڈی ڈاکٹر موجود نہیں  10. وقت بہت زیادہ تھا  11. جانے کی اجازت نہیں ملی  12. دیگر ، وضاحت | آپ نے مضر اثرات کی صوررت میں کوئی طبی مشورہ / علاج کیوں نہیں حاصل کیا ؟ | **CU15** |
|  | | 1. ہاں  2. نہیں | کیا آپ اپنے موجودہ طریقے سے جو آپ آپ استعمال کررہے ہیں مطمئن ہیں؟ | **CU16** |
|  | | 1. ہاں  2. نہیں | کیا آپ اس سے اور موئثر طریقہ چاہتے ہیں؟ | **CU17** |
|  | |  | اگر ہاں تو کیوں؟ | **CU17A** |
|  | |  | اگر نہیں تو کیوں؟ | **CU17B** |
| **CU18A** اگر نہیں تو  پر جائیں | | 1. ہاں  2. نہیں | کیا آپ کو کبھی بھی کوئی طریقہ استعمال کرنے کے دوران حمل ہوا؟ | **CU18** |
|  | | 1. استعمال کا طریقہ معلوم نہیں تھا  2. دوس بھول گئی  3. طریقہ کام نہیں کر پایا  12. دیگر ، وضاحت ـــــــــــــــــــــــــ | اگر ہاں تو اُ س کی کیا وجہ ہے؟ | **CU18A** |
|  | | 1. ہاں  2. نہیں  99. یاد نہیں | جب آپ نے موجودہ طریقہ حاصل کیا تو کیا اُس وقت آپ کو دوسرے طریقوں کے بارے میں بتایا گیا تھا جو آپ استعمال کرتی تھی ؟ | **CU19** |
|  | | **پبلک سیکٹر**  1. گورنمنٹ / آر ایچ ایس اے  2. رورل ہیلتھ سینٹر  3. فیملی ویلفئیر سینٹر  4. ایم سی ایچ  5. ڈسپینسری  6. موبائل سروس کیمپ  7. لیڈی ہیلتھ ورکر  8. لیڈی ہیلتھ وزیٹر  9. بیسک ہیلتھ یونٹ  10. میل موبلا ئزر  11. ایف ڈبلیو اے  96. دیگر ، پبلکــــــــــــــــــــــــــــــ  **پرائیوٹ /این جی او میڈیکل سیکٹر**  12. فارمیسی ، کیمسٹ  13. پرائیوٹ ڈاکٹر  14. ہومیو پیتھک  15. ڈسپینسر / کمپائو نڈر  16. امن ہیلتھ ورکر  96. دیگر پرائیوٹ میڈیکل ـــــــــــــــــ  17. دکان (فارمیسی اور ڈسپیسری کے علاوہ)  18. دوست / رشتہ دار  19. حکیم  20. دائی ، ٹریڈ برتھ اٹینڈنٹ  96. دیگر وضاحت ــــــــــــــــــــــــ  98. نہیں معلوم  99. یاد نہیں | آپ نے موجودہ طریقہ (زیر استعمال طریقہ) آخری بار کہاں سے حاصل کیا ؟ | **CU20** |
| **CU24** اگر نہیں تو  پر جائیں | | 1. ہاں  2. نہیں | کیا ان 6 مہینوں میں کوئی ہیلتھ ورکر نے آپ کے یہاں وزٹ کیا؟ | **CU21** |
|  | | 1. گورنمنٹ  2. سکھ / امن فاؤنڈیشن  3. دونوں  96. اور کوئی  98. نہیں معلوم  99. یاد نہیں | کیا آپ بتاسکتی ہیں کہ ہیلتھ ورکر کا تعلق کس ادارے سے تھا ؟ | **CU22** |
|  | | 1. ماں اور بچے کی صحت  2. خاندانی منصوبہ بندی  3. مانع حمل طریقوں کا سامان  4. فیملی پلاننگ سینٹر یا کلینک کے حوالے کردیا  5. مضر اثرات کا علاج مہیا کرنا  6. بچوں کے حفاظتی ٹیکے  7. معمولی تکالیف کا علاج مہیا کرنا  8. تیلی ہیلتھ کی سہولت کے بارے میں آگاہی مہیا کرنا  9. خا ندانی زندگی کی تعلیم کے متعلق آگاہی مہیا کر نا  10. خاندانی منصوبہ بندی سے متعلق معلومات  96. اور کوئی  98. نہیں معلوم  99. یاد نہیں | آپ کو ہیلتھ ورکر سے کیا معلومات حاصل ہوئی ؟ | **CU23** |
| ایل ایچ ڈبلیو | امن (سی ایچ ڈبلیو) | 1. ماں اور بچے کی صحت سے متعلق آگاہی  2. بچوں کے حفاظتی ٹیکے  3. فیملی پلاننگ کے متعلق قائل کرتی ہیں  4. خاندانی منصوبہ بندی کے لئے استعمال ہونے والی گولیا ں / کنڈم دیتی ہیں  5. مضر اثرات کا علاج مہیا کیا  6. فیملی پلاننگ سینٹر / ہیلتھ سینٹر کے حوالے کردیا  7. معمولی تکالیف کا علاج مہیا کیا  8. (ملاقات کا دورانیہ)  9. دوائیوں کی فراہمی  10. دودھ پلانے کے متعلق معلومات  11. زچگی کے بعد کی دیکھ بھال کے متعلق آگاہی  12. حمل ضائع ہونے کے بعد کی دیکھ بھال کے متعلق آگاہی  13. بچے اور ماں کی غذائیت  14. کمیونٹی ہیلتھ ورکر کے رویے سے مطمئن ہیں  15. ہیلتھ فیسیلٹی میں اپنے ساتھ لے گئےس  16. بی پی چیک کرتی ہیں  17. ماں کا وزن چیک کرتی ہیں  18. پانچ سال سے کم بچوں کا وزن چیک کرتی ہیں  96. اور کوئی  98. نہیں معلوم  99. یاد نہیں | **کیا آپ ہیلتھ ورکر کی دی گئی معلومات سے مطمئن ہیں؟** | **CU23A** |
| **CU25** اگر نہیں تو  پر جائیں | | 1. ہاں  2. نہیں | کیا پچھلے 6 مہینو ں کے دوران آپ نے اپنی یا اپنے بچوں کے علاج کے لئے کسی طبی سہولت / ادارے کا دورہ کیا ہے؟ | **CU24** |
|  | | 1. گھر پر سہولات فراہم کی جاتی ہے  2. دوسری سہولت فراہم کرنے والو کے پاس جانا پسند کرتے ہیں  3. جگہ مناسب نہیں ہے  4. سینٹر کا دورہ کرنے کی ضرورت پیش نہیں آئی  5. سینٹر کی موجودہ سہولتوں کے بارے میں معلومات نہیں  6. مزید بچے چاہئے  96. اور کوئی  98. نہیں معلوم  99. یاد نہیں | اگر نہیں تو طبی سہولت /ادارے کا دورہ نہ کرنے کی کیا وجہ ہے؟ | **CU25** |

***خاندانی منصوبہ بندی کے متعلق معلومات اور اُ س کے استعمال سے متعلق سوالات***

| **جوابات** | **CONDOMS (CD)** | |  |
| --- | --- | --- | --- |
| **CD3** اگر نہیں اور معلوم نہیں تو  پر جائیں | 1. ہاں  2. نہیں  98. نہیں معلوم  96. دیگر وضاحت ــــــــــــــــــــــــ | کیا آپ کے خیال میں کونڈم (ربڑ) استعمال کرنے کے باوجود حمل ہوسکتا ہے ؟ | **CD1** |
|  | 1. اگر صحیح طرح نہ پہنا جائے  2. اگر ربڑ اتارتے وقت مرد کا پانی عورت کے اندر چلا جائے  3. اگر استعمال کے دوران (تعلق کے دوران) ربڑ پھٹ جائے  98. نہیں معلوم  99. یاد نہیں  96. دیگر وضاحت ــــــــــــــــــــــــ | اگر ہاں تو اس کیا وجوہات ہوسکتی ہیں ؟ | **CD2** |
|  | 1. بے حد موئثر ہے  2. موئثر ہے  3. زیادہ موئثر نہیں  4. موئثر نہیں ہے  98. نہیں معلوم  99. یاد نہیں  96. دیگر وضاحت ــــــــــــــــــــــــ | آپ کے خیال میں کونڈم (ربڑ) کا استعمال حمل کو روکنے کے لئے کتنا مئو ثر ہے؟ | **CD3** |
|  | 1. ہاں  2. نہیں  98. نہیں معلوم  99. یاد نہیں  96. دیگر وضاحت ــــــــــــــــــــــــ | کیا آپ کے علاقے میں شادی شدہ جوڑوں کو (کونڈم) ربڑ آسانی سے مل جاتا ہے ؟ | **CD4** |
|  | 1. ہاں  2. نہیں  3. لا گو نہیں  98. نہیں معلوم  99. یاد نہیں  96. دیگر وضاحت ــــــــــــــــــــــــ | کیا آپ کے خیال میں آپ کے علاقے کے لوگ کنڈم (ربڑ) آسانی سے خرید سکتے ہیں ؟ | **CD5** |

|  | 1. کم قیمت / سستہ طریقہ ہے  2. آسانی سے مل جاتا ہے  3. جنسی بیماریوں کو روکتا ہے  4. استعمال میں آسان ہے  5. مضر اثرات نہیں ہوتے  98. نہیں معلوم  99. یاد نہیں  96. دیگر وضاحت ــــــــــــــــــــــــ | کو ئی طریقہ استعمال کرنے کی بنیادی وجہ کیا ہے؟ | **CD6** |
| --- | --- | --- | --- |
|  | 1. مرد کو الرجی /خارش کرسکتا ہے  2. عورت کو الرجی /خارش کرسکتا ہے  3. جنسی تعلقات کی لذت کم ہوجاتی ہے  4. کونڈم (ربڑ) لیتے ہوئے شرم آتی ہے  5. استعمال کے بعد ضائع کرنا / پھینکنا مشکل ہے  6. طریقہ پسند نہیں  98. نہیں معلوم  99. یاد نہیں  96. دیگر وضاحت ــــــــــــــــــــــــ | ایسی کیا ایک چیز ہے جو آپ کو اس طر یقے سے متعلق پسند نہیں؟ | **CD7** |
| **CONTRACEPTIVE PILLS (OCP)** | | | |
|  | 1. ایک گولی روزانہ  2. شوہر سے تعلق کے وقت  3. ہفتے میں ایک دفعہ  98. نہیں معلوم  99. یاد نہیں  96. دیگر وضاحت ــــــــــــــــــــــــ | مانع حمل اور وقفے کی گولی کس طرح لی جاتی ہے؟ | **OCP1** |
|  | 1. ہاں  2. نہیں  98. نہیں معلوم  96. دیگر وضاحت ــــــــــــــــــــــــ | کیا آپ کے خیال میں وقفے کی گولی استعمال کی کے باوجود حمل ہوسکتا ہے؟ | **OCP2** |
|  | 1. بے حد موئثر ہے  2. موئثر ہے  3. زیادہ موئثر نہیں  4. موئثر نہیں ہے  98. نہیں معلوم  99. یاد نہیں  96. دیگر وضاحت ــــــــــــــــــــــــ | آپ کے خیال میں وقفے کی گولی کا استعمال بچوں میں وقفے کے لئے کتنا مو ئثر ہے ؟ | **OCP3** |
|  | 1. ہاں  2. نہیں  98. نہیں معلوم  99. یاد نہیں  96. دیگر وضاحت ــــــــــــــــــــــــ | کیا آپ کے علاقے میں وقفے کی گولیا ں آسانی سے دستیاب ہیں؟ | **OCP4** |
|  | 1. ہاں  2. نہیں  98. نہیں معلوم  99. یاد نہیں  96. دیگر وضاحت ــــــــــــــــــــــــ | کیا آپ کے خیال میں آپ کے علاقے کے لوگ وقفے کی گولیا ں آسانی سے خرید سکتی ہیں ؟ | **OCP5** |
|  | 1. کم قیمت  2. آسانی سے مل جاتا ہے  3. مضر اثرات نہیں ہوتے  4. عورت استعمال کرسکتی ہے شوہر کے علم کے بغیر  5. استعمال میں آسان ہے  6.  7. عارضی طریقہ  98. نہیں معلوم  99. یاد نہیں  96. دیگر وضاحت ــــــــــــــــــــــــ | مانع حمل / وقفے کوگولی کے طریقے کو منتخب کرنے کی بنیادی وجہ کیا ہے ؟ | **OCP6** |
|  | 1. ماہواری میں خون کا زیادہ بہنا  2. گولی کا استعمال روکنے پر خون کا آنا  3. ماہواری میں درد ہونا  4. وزن کا بڑھنا  5. متلی اور الٹی کا آنا  6. روزانہ کھانی پڑتی ہے  7. طریقے کے بارے میں معلوم نہیں  98. نہیں معلوم  99. یاد نہیں  96. دیگر وضاحت ــــــــــــــــــــــــ | اس طریقے سے متعلق ایسی کیا چیز ہے جو آپ کو پسند نہیں؟ | **OCP7** |
| **Injection** | | | |
|  | 1. ہر 3-2 مہینے  2. ہر مہینے  98. نہیں معلوم  99. یاد نہیں  96. دیگر وضاحت ــــــــــــــــــــــــ | آپ کے خیال میں بچوں کی پیدائش میں وقفے کے لئے ٹیکے کتنے عرصے بعد لگائے جاتے ہیں ؟ | **INJ1** |
|  | 1. بے حد موئثر ہے  2. موئثر ہے  3. زیادہ موئثر نہیں  4. موئثر نہیں ہے  98. نہیں معلوم  99. یاد نہیں  96. دیگر وضاحت ــــــــــــــــــــــــ | آپ کے خیال میں مانع حمل ٹیکوں / وقفے کے ٹیکوں کا استعمال بچوں میں وقفے کے لئے کتنا مو ثئر ہے ؟ | **INJ2** |
|  | 1. ہاں  2. نہیں  98. نہیں معلوم  99. یاد نہیں  96. دیگر وضاحت ــــــــــــــــــــــــ | آپ کے خیال میں ٹیکہ استعمال کرنے کے باوجود حمل ہوسکتا ہے ؟ | **INJ3** |
|  | 1. ہاں  2. نہیں  98. نہیں معلوم  99. یاد نہیں  96. دیگر وضاحت ــــــــــــــــــــــــ | کیا آپ کے علاقے میں مانع حمل / وقفے کے ٹیکے آسانی سے دستیاب ہیں ؟ | **INJ4** |
|  | 1. ہاں  2. نہیں  98. نہیں معلوم  99. یاد نہیں  96. دیگر وضاحت ــــــــــــــــــــــــ | کیا آپ کے علاقے کے لوگ مانع حمل اور وقفے کے ٹیکے آسانی سے خرید سکتے ہیں ؟ | **INJ5** |
|  | 1. روزانہ نہیں لینا پڑتا  2. دودھ پلانے والی مائیں بھی استعمال کرسکتی ہیں  3. 3-2 سے مہینے تک حمل سے محفوظ رکھتا ہے  4. کسی بھی وقت استعمال چھوڑا جاسکتا ہے  5. خفیہ طور پر استعمال کیا جاسکتا ہے  6. آسانی سے مل جاتا ہے  7. کم قیمت / سستا طریقہ  8. جنسی تعلقات پر اثر انداز نہیں ہوتا  98. نہیں معلوم  99. یاد نہیں  96. دیگر وضاحت ــــــــــــــــــــــــ | اس طریقے کو پسند کرنے کی بنیادی وجہ کیا ہے؟ | **INJ6** |
|  | 1. وقت یاد رکھنا مشکل ہے  2. ہر 3-2 مہینے بعد ٹیکا دوبارہ لگوانا پڑتا ہے  3. ماہواری میں خون زیادہ آتا ہے  4. دو ماہواری کے درمیان خون کا آنا  5. ماہواری میں درکار ہونا  6. وزن کا بڑھنا  7. ماہواری کا بند ہوجانا / نہ آنا  8. متلی اور الٹی کا آنا  98. نہیں معلوم  99. یاد نہیں  96. دیگر وضاحت ــــــــــــــــــــــــ | اس طریقے سے متعلق کیا چیز ہے جوآپ کو پسند نہیں؟ | **INJ7** |
| **IUCD (IUD)** | | | |
|  | 1. بچہ دانی میں  2. اندام دہانی میں  3. پیٹ کے اندر  98. نہیں معلوم  99. یاد نہیں  96. دیگر وضاحت ــــــــــــــــــــــــ | آپ کے خیال میں (چھلہ) عورت کے جسم کے کس حصے میں رکھاجاتا ہے؟ | **IUD1** |
|  | 5.1 سال تک  10.2 سال تک  3.3-2 سال تک  4.وقت کی کوئی حد نہیں | چھلہ استعمال کرنے کی صورت میں بچوں کی پیدائش میں کتنا وقفہ کیا جاسکتا ہے ؟ | **IUD2** |
|  | 1. ہاں  2. نہیں  98. نہیں معلوم  99. یاد نہیں  96. دیگر وضاحت ــــــــــــــــــــــــ | آپ کے خیال میں (آئی یو ڈی) استعمال کرنے کے باوجود حمل ہوسکتا ہے؟ | **IUD3** |
|  | 1. بے حد موئثر ہے  2. موئثر ہے  3. زیادہ موئثر نہیں  4. موئثر نہیں ہے  98. نہیں معلوم  99. یاد نہیں  96. دیگر وضاحت ــــــــــــــــــــــــ | آپ کے خیال میں بچوں میں وقفے کے لئے چھلہ کا استعمال کتنا مو ثئر ہے ؟ | **IUD4** |
|  | 1. ہاں  2. نہیں  98. نہیں معلوم  99. یاد نہیں  96. دیگر وضاحت ــــــــــــــــــــــــ | کیا آپ کے علاقے میں چھلہ آسانی سے دستیاب ہے؟ | **IUD5** |
|  | 1. ہاں  2. نہیں  98. نہیں معلوم  99. یاد نہیں  96. دیگر وضاحت ــــــــــــــــــــــــ | کیا آپ کے علاقے میں لوگ چھلہ آسانی سے خرید سکتے ہیں؟ | **IUD6** |
|  | 1. ہر ماہواری کے بعد مہینے میں ایک دفعہ  2. ہفتے میں ایک دفعہ  3. ہر تین مہینے میں  4. جب عورت چاہے چیک کرسکتی ہے  98. نہیں معلوم  99. یاد نہیں  96. دیگر وضاحت ــــــــــــــــــــــــ | چھلہ استعمال کرنے والی عورت کو چھلے کا دھاگہ کتنے عرصے میں چیک کرنا  چاہئے؟ | **IUD7** |
|  | 5.1-2 سال تک وقفہ دینا ہے  2. دودھ پلانے والی مائیں بھی استعمال کرسکتی ہیں  3. کسی بھی وقت استعمال ترک کیا جاسکتا ہے  4. خوراک بھولنے کا ڈر نہیں گولی / ٹیکے کی طرح  5. مضر اثرات نہیں  6. جنسی تعلقات پر اثر انداز نہیں ہونا  98. نہیں معلوم  99. یاد نہیں  96. دیگر وضاحت ــــــــــــــــــــــــ | اس طریقے کو پسند کرنے کی ایک بنیادی وجہ کیا ہے؟ | **IUD8** |
|  | 1. ماہواری میں خون کا زیادہ بہنا  2. استعمال روکنے پر خون کا زیادہ آنا  3. ماہواری میں درد کا ہونا  4. جنسی تعلق کے وقت درد ہونا  5. جنسی تعلق کے وقت شوہر کو تکلیف ہونا  6. چھلہ کا بچہ دانی سے باہر آجانا  7. سوجن اور انفیکشن کا ہونا  8. بچہ دانی کا کینسر ہونا  9. چھلہ کا بچہ دانی کے پھٹو ں میں چلے جانا  98. نہیں معلوم  99. یاد نہیں  96. دیگر وضاحت ــــــــــــــــــــــــ | اس طریقے سے متعلق کیا چیز ہے جو آپ کو پسند نہیں؟ | **IUD9** |
| **IMPLANT (IP)** | | | |
|  | 1. بائیں بازوں کے اوپری حصے میں  2. بچے دانی کے اندر  98. نہیں معلوم  99. یاد نہیں  96. دیگر وضاحت ــــــــــــــــــــــــ | آپ کے خیال میں کیپسول (امپلانٹ) کہاں لگایا جاتا ہے؟ | **IP1** |
|  | 5.1سا ل  10.2 سا ل  3.3 مہینے  3.4-2 سا ل  98. نہیں معلوم  99. یاد نہیں  96. دیگر وضاحت ــــــــــــــــــــــــ | 1. کیا آپ جانتی ہیں کہ کیپسول کا استعمال کتنے عرصے تک وقفہ مہیا کر سکتا ہے ؟ | **IP2** |
|  | 1. بے حد موئثر ہے  2. موئثر ہے  3. زیادہ موئثر نہیں  4. موئثر نہیں ہے  98. نہیں معلوم  99. یاد نہیں  96. دیگر وضاحت ــــــــــــــــــــــــ | آپ کے خیال میں کیپسول (امپلانٹ) حمل میں وقفے کے لئے کتنا مو ثر ہے ؟ | **IP3** |

|  | 1. ہاں  2. نہیں  98. نہیں معلوم  99. یاد نہیں  96. دیگر وضاحت ــــــــــــــــــــــــ | کیا آپ کے خیال میں امپلانٹ استعمال کرنے کے باوجود حمل ہوسکتا ہے؟ | **IP4** |
| --- | --- | --- | --- |
|  | 1. ہاں  2. نہیں  98. نہیں معلوم  99. یاد نہیں  96. دیگر وضاحت ــــــــــــــــــــــــ | کیا آپ کے علاقے میں کیپسول (امپلانٹ) آسانی سےدستیاب ہیں ؟ | **IP5** |
|  | 1. ہاں  2. نہیں  98. نہیں معلوم  99. یاد نہیں  96. دیگر وضاحت ــــــــــــــــــــــــ | کیا آپ کے علاقے کے لوگ کیپسول (امپلانٹ) آسانی سے خرید سکتے ہیں ؟ | **IP6** |
|  | 5.1 سال کے لئے وقفہ مہیا کرتی ہے  2. دودھ پلانے والی مائیں بھی استعمال کرتی ہیں  3. مضر اثرات نہیں  98. نہیں معلوم  99. یاد نہیں  96. دیگر وضاحت ــــــــــــــــــــــــ | پیدائش میں وقفے کے اس طریقے کو پسند کرنے کی بنیادی وجہ کیا ہے؟ | **IP7** |
|  | 1. ماہواری میں خون کا زیادہ بہنا  2. دو ماہواری کے درمیان خون کا آنا  3. ماہواری میں درد ہونا  3. کیپسول کا اپنی جگہ سے نکل جانے کا ڈر  4. سوجن اور انفیکشن  5. پانی کا آنا  98. نہیں معلوم  99. یاد نہیں  96. دیگر وضاحت ــــــــــــــــــــــــ | اس طریقے سے متعلق ایسی کیا چیز ہے جو آپ کو پسند نہیں؟ | **IP8** |

| **جوابات** | **Section F: Discontinuation of Contraceptive Use** | |  |
| --- | --- | --- | --- |
| **اب میں آپ سے خاندانی منصوبہ بندی کے طریقوں کے استعمال سے متعلق سوال کروں گی جن کو بند کئے ہوئے چھ ماہ ہوگئے ہیں۔** | | | |
|  | مہینہ  سال | کونسے مہینے اور سال سے آپ نے بچوں کی پیدائش کے وقفے کے طریقے کو روک دیا ہے؟ | **DC1** |
|  | 1. انجیکشن  2. امپلانٹ  3. فیمیل کنڈم  4. آئی یو ڈی  96. دیگر وضاحت ــــــــــــــــــــــــ | وہ طریقہ کون سا تھا جو آپ نے چھوڑ دیا؟ | **DC2** |
|  | 1. رسائی کی کمی  2. تر جیحی طریقہ دستیاب نہیں  3. استعمال کرنے میں آسان نہیں  4. جسم کے معمول کے عمل کے ساتھ مداخلت کرتا ہے  5. مضر اثرات  6. شوہر سے ملاپ بہت کم ہے  7. شوہر سے ملاپ نہیں ہے  8. خدا کے حوالے  9. بچے کو دودھ پلاتی ہوں  10. مضر اثرات کا خوف  11. شوہر مخالف ہے  12. مذہب کی ممانعت  13. دوست مخالف ہے /پڑوسی مخالف ہے/ رشتہ دار مخالف ہے  14. حاملہ بننا چاہتی تھی  96. دیگر وضاحت ــــــــــــــــــــــــ | آخری طریقہ چھوڑنے کی بنیادی وجہ کیا تھی؟ | **DC3** |
|  |  | آخری طریقے کو چھوڑنے کی بنیادی وجہ کیا ہے؟ | **DC4** |
|  | 1. مرد کو الرجی /خارش کرسکتا ہے  2. عورت کو الرجی /خارش کرسکتا ہے  3. جنسی تعلقات کی لذت کم ہوجاتی ہے  4. کونڈم (ربڑ) لیتے ہوئے شرم آتی ہے  5. استعمال کے بعد ضائع کرنا / پھینکنا مشکل ہے  6. طریقہ پسند نہیں  98. نہیں معلوم  99. یاد نہیں  96. دیگر وضاحت ــــــــــــــــــــــــ | کونڈم کو چھوڑنے کی بنیادی وجہ کیا ہے؟ | **DC4.1** |
|  | 1. ماہواری میں خون کا زیادہ بہنا  2. گولی کا استعمال روکنے پر خون کا آنا  3. ماہواری میں درد ہونا  4. وزن کا بڑھنا  5. متلی اور الٹی کا آنا  6. روزانہ کھانی پڑتی ہے  7. طریقے کے بارے میں معلوم نہیں  98. نہیں معلوم  99. یاد نہیں  96. دیگر وضاحت ــــــــــــــــــــــــ | وقفے کی گولی کو چھوڑنے /بند کرنے کی بنیادی وجہ کیا ہے؟ | **DC4.2** |
|  | 1. وقت یاد رکھنا مشکل ہے  2. ہر 3-2 مہینے بعد ٹیکا دوبارہ لگوانا پڑتا ہے  3. ماہواری میں خون زیادہ آتا ہے  4. دو ماہواری کے درمیان خون کا آنا  5. ماہواری میں درکار ہونا  6. وزن کا بڑھنا  7. ماہواری کا بند ہوجانا / نہ آنا  8. متلی اور الٹی کا آنا  98. نہیں معلوم  99. یاد نہیں  96. دیگر وضاحت ــــــــــــــــــــــــ | مانع حمل ٹیکوں کے استعمال کو چھوڑنے کی بنیادی وجہ کیا ہے | **DC4.3** |
|  | 1. ماہواری میں خون کا زیادہ بہنا  2. استعمال روکنے پر خون کا زیادہ آنا  3. ماہواری میں درد کا ہونا  4. جنسی تعلق کے وقت درد ہونا  5. جنسی تعلق کے وقت شوہر کو تکلیف ہونا  6. چھلہ کا بچہ دانی سے باہر آجانا  7. سوجن اور انفیکشن کا ہونا  8. بچہ دانی کا کینسر ہونا  9. چھلہ کا بچہ دانی کے پھٹو ں میں چلے جانا  98. نہیں معلوم  99. یاد نہیں  96. دیگر وضاحت ــــــــــــــــــــــــ | آئی یو سی ڈی (چھلہ) کو چھوڑنے /بند کرنے کی بنیا دی وجہ کیا ہے؟ | **DC4.4** |
|  | 1. ماہواری میں خون کا زیادہ بہنا  2. دو ماہواری کے درمیان خون کا آنا  3. ماہواری میں درد ہونا  3. کیپسول کا اپنی جگہ سے نکل جانے کا ڈر  4. سوجن اور انفیکشن  5. پانی کا آنا  98. نہیں معلوم  99. یاد نہیں  96. دیگر وضاحت ــــــــــــــــــــــــ | امپلانٹ کو چھوڑنے کی بنیادی وجہ کیا ہے؟ | **DC4.5** |
|  | 1. بنیادی طور پر میرا  2. بنیادی طور پر شوہر کا  3. مل کے فیصلہ  96. دیگر وضاحت کریں | حمل کو روکنے یا بچوں میں وقفہ کے خاندانی منصوبہ بندی کے طریقوں کو چھوڑنے / بند کرنے کا بنیادی فیصلہ آپ کا ہے یا آپ کے شوہر کا یا آپ دونوں کا؟ | **DC5** |
|  | 1. ہاں  2. نہیں | کیا آپ روایتی طریقوں کو ترجیح دیتی ہے جدید طریقوں پر؟ | **DC6** |
|  | 1. ہاں  2. نہیں | کیا آپ کو جدید طریقہ مستقبل کے استعمال کے لئےکافی مہنگا لگتا ہے؟ | **DC7** |
|  | 1. ہاں  2. نہیں | کیا وہ ذرائع یا نجی یا عوامی جہاں سے آپ جدید طریقہ حاصل کرتی ہے بہت دور ہے؟ | **DC8** |
|  | 1. ہاں  2. نہیں | کیا آپ کی نظر میں یہ ٹھیک ہے کہ کسی بھی طریقے کو وقتی طور پر چھوڑدیان چاہئے اور پھر دوبارہ شروع کرنا چاہئے؟ | **DC9** |
|  | 1. ہاں  2. نہیں | کیا آپ مستقبل میں بچے کی پیدائش میں یا مانع حمل کے لئے جدید طریقے کے استعمال کی طرف جائیں گے؟ | **DC10** |
