# Supplementary material for: Factors associated with the discontinuation of modern methods of contraception in the low income areas of Sukh Initiative Karachi: A community-based case control study
Source: PLoS One. 2019 Jul 3;14(7):e0218952. doi: 10.1371/journal.pone.0218952 (PMC6608957; doi:10.1371/journal.pone.0218952)
Supplement: S2 File — (DOC) [file pone.0218952.s002.doc]

FACTORS ASSOCIATED WITH DISCONTINUATION OF MODERN METHODS OF CONTRACEPTION AMONG WOMEN OF REPRODUCTIVE AGE GROUP IN LOW INCOME AREAS OF KARACHI

AGA KHAN UNIVERSITY HOSPITAL

**QUESTIONNAIRE**

| **Married Woman`s Questionnaire** | |
| --- | --- |
| **Town’s Name** | **Korangi Malir Bin-Qasim Landhi** |
| **Area name** |  |
| **Household Number** |  |
| **Woman`s Name** |  |
| **Husband’s Name** |  |
| **Phone number** |  |
| **Name of interviewer** |  |
| **Date of interview** | **Day____ Month______ 2014** |
| **Name of Field Supervisor** |  |
| **Field data editing date** |  |
| **Desk editor** |  |
| **Data entry date** |  |
| **Status of the interview**  01.Completed  02.Incomplete  03.Not at home  04.Refused  05.Household not found |  |

| **Section: B Respondent’s Background Characteristics** | | | **Response** |
| --- | --- | --- | --- |
| **RB1.** | How old are you? (in completed years) | **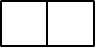** |  |
| **RB2.** | Is your husband living with you now or is he staying elsewhere | 1.Living with him  2.Staying elsewhere |  |
| **RB3.** | Duration of current marriage  (in completed years) | **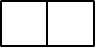** |  |
| **RB4.** | What was your age at first marriage in completed years) | **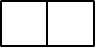** |  |
| **RB5.** | Have you ever attended a formal school? | 1.Yes  2. No |  |
| **RB6.** | What is the highest class you have completed? | 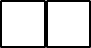 |  |
| **RB7.** | Do you read a newspaper or magazine daily, at least once a week, occasionally or not at all? | 1.Daily  2.At least once a week  3.Occasionally  4.Not at all |  |
| **RB8.** | Do you listen to the radio daily, at least once a week, occasionally or not at all? | 1.Daily  2. At least once a week  3. Occasionally  4.Not at all |  |
| **RB9.** | Do you watch television daily, at least once a week, occasionally or not at all? | 1.Daily  2. At least once a week  3. Occasionally  4.Not at all |  |
| **RB10.** | Do you own a mobile phone? | 1.Yes  2. No | **If Yes Go to RB13** |
| **RB11.** | If No, do you use someone else`s phone? | 1.Yes  2. No | **If No go to RB14** |
| **RB12.** | If Yes, whose mobile phone do you usually use? | 1.Husband’s  2. Neighbours  3. Friends  96.Any other ___________ (specify) |  |
| **RB13.** | Do you use Mobile phone daily, at least once a week, occasionally or not at all? | 1.Daily  2. At least once a week  3. Occasionally  4.Not at all |  |
| **RB14.** | What is your mother tongue? | 1.Urdu  2.Punjabi  3.Sindhi  4.Pushto  5.Balochi  6.English  7.Barauhi  8.Siraiki  9. Hindko  10.Kashmiri  11.Pahari  12. Potohwari  13.Marwari  14. Farsi  96.Other (Specify) |  |

| **Section: C Socio-demographic Information** | | | **Response** |
| --- | --- | --- | --- |
| **SD1.** | What is the ownership status of your house? | 1. Owned 2. Rented   96.Other (specify) |  |
| **SD2.** | How many rooms are there in the household? (excluding toilet, kitchen, and garage) | 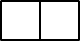 |  |
| **SD3.** | Are you working currently for  earning money? | 1.Yes  2. No  96.Others (specify) | **If No Go to SD6.** |
| **SD4.** | What type of work you do? | 1.Farm work  2.Teacher  3.House maid  4.Lady health  worker\Nurse\ Health care  worker  96.Others (specify ) |  |
| **SD5.** | Were you working to earn income before getting married? | 1.Yes  2. No |  |
| **SD6.** | Whenever you get sick who  makes decision for you to seek treatment?  More than one responses are possible | 1.Respondent herself  2.Husband  3.Mother in law  4.Father in law  96.Others (specify)------ |  |
| **SD7.** | Does your husband work to earn money? | 1.Yes  2. No | **If No Go to SD9.** |
| **SD8.** | If yes then what is his occupation? | 1.Farmer  2.Fisherman  3.Teacher  4.Work on Hotel  5.Labourer  6.Shopkeeper  96.Others specify |  |
| **SD9.** | What is the total household income? | In Rupees |  |
| **SD10.** | Does your household have :  **Encircle the item possessed by household**  (write actual numbers) | 1.Electricity  2. Radio  3. Television  4. Landline telephone  5.Refrigerator  6. Almirah/cabinet  7. Chair  8. Room cooler  9. Air conditioner  10.Washing machine  11.Water pump  12. Bed  13. Clock  14. Sofa  15.Camera  16. Sewing machine  17. Computer  18. Internet connection  19.None of the above mentioned items | If Yes Write  down the  number |
| **SD11.** | Does any member of this household own:  **Encircle the item possessed by household**  (write actual numbers) | 1.Watch  2.A mobile telephone  3.Bicycle  4.Motorcycle/scooter  5. Animal-drawn cart car/truck/bus  6.Tractor  7.Boat with motor  8.Boat without a MOTOR  9. None of the above | If Yes Write  down the  number |
| **SD12.** | Does any member of this household own any agricultural land? | 1. Yes 2. No | **If yes then how many acres.(Write actual land in acres)** |
| **SD13.** | Do you possess any of these  livestock’s? (write actual  numbers in response column) | 1.Cow  2.Buffalo  3.Goat  4.Bull/Ox  5.Sheep  6.Camel  7.Hens/Cock  8.None of the above mentioned animals |  |

| **Section:D Reproductive Health** | | | **Response** |
| --- | --- | --- | --- |
| Now I would like to ask you about all the pregnancies that you have had during your life. By this I mean all the children born to you whether they were born alive or dead, whether they are still living or not, whether they live with you or somewhere else | | | |
| **RH1.** | Have you ever given birth? | 1.Yes  2. No | **If No, go to RH18** |
| **RH2.** | Do you have any sons or daughters to whom you have given birth who are now living with you? | 1.Yes  2. No |  |
| **RH3.** | Number of living children? | 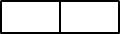 |  |
| **RH4.** | 1. How many sons live with you? 2. How many daughters live with you?   **If none, Record '00** | a. Sons at  home   1. Daughters   at home |  |
| **RH5.** | Do you have any sons or daughters to whom you have given birth who are alive but do not live with you? | 1.Yes  2. No | **If No, go to RH7** |
| **RH6.** | 1. How many sons are alive but do not live with you? 2. How many daughters are alive but do not live with you?   **If none, Record '00'** | 1. Sons   elsewhere   1. b. Daughters   elsewhere |  |
| **RH7.** | Have you ever given birth to a boy or girl who was born alive but later died? | 1.Yes  2. No | **If No, go to RH9** |
| **RH8.** | a. How many boys have died?  b. How many girls have died?  **If none, Record '00'** | a. Sons died  b. Daughters  died |  |
| **RH9.** | Have you ever had a pregnancy that did not end in a live birth or resulted in a miscarriage, abortion or still birth? | 1.Yes  2. No | **If No, go to RH11** |
| **RH10.** | How many pregnancies have you had that did not end in a live birth? | 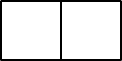 |  |
| **RH11.** | How many antenatal visits a woman should have during her pregnancy? | 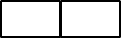 |  |
| **RH12.** | How many antenatal visits you had when you were pregnant with your last child? | 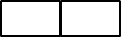 |  |
| **RH 13.**  **Now I would like to record all your pregnancies, whether born alive, born dead, or lost before full term, starting with the first one you had.** | | | |

**
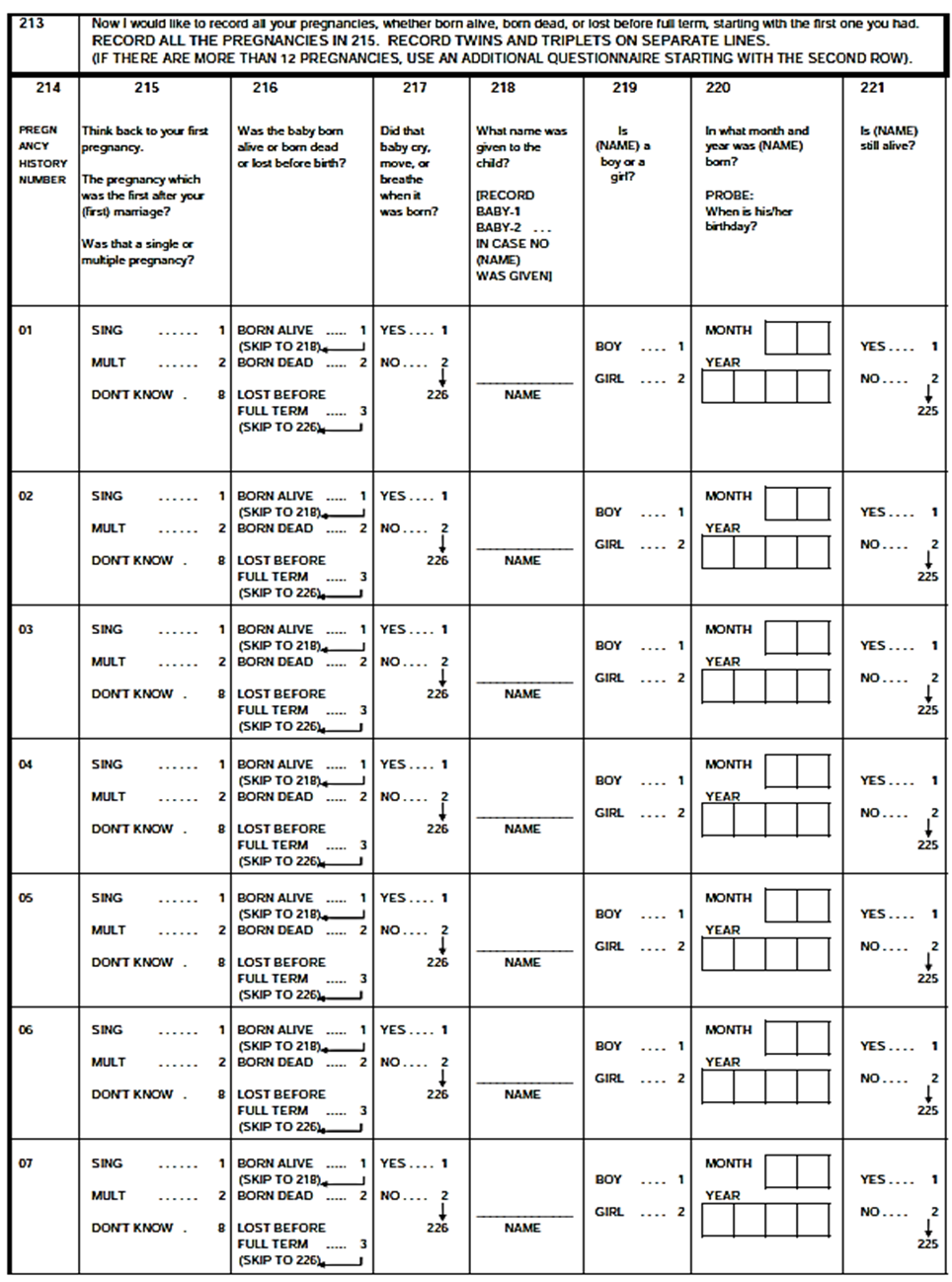
**

**
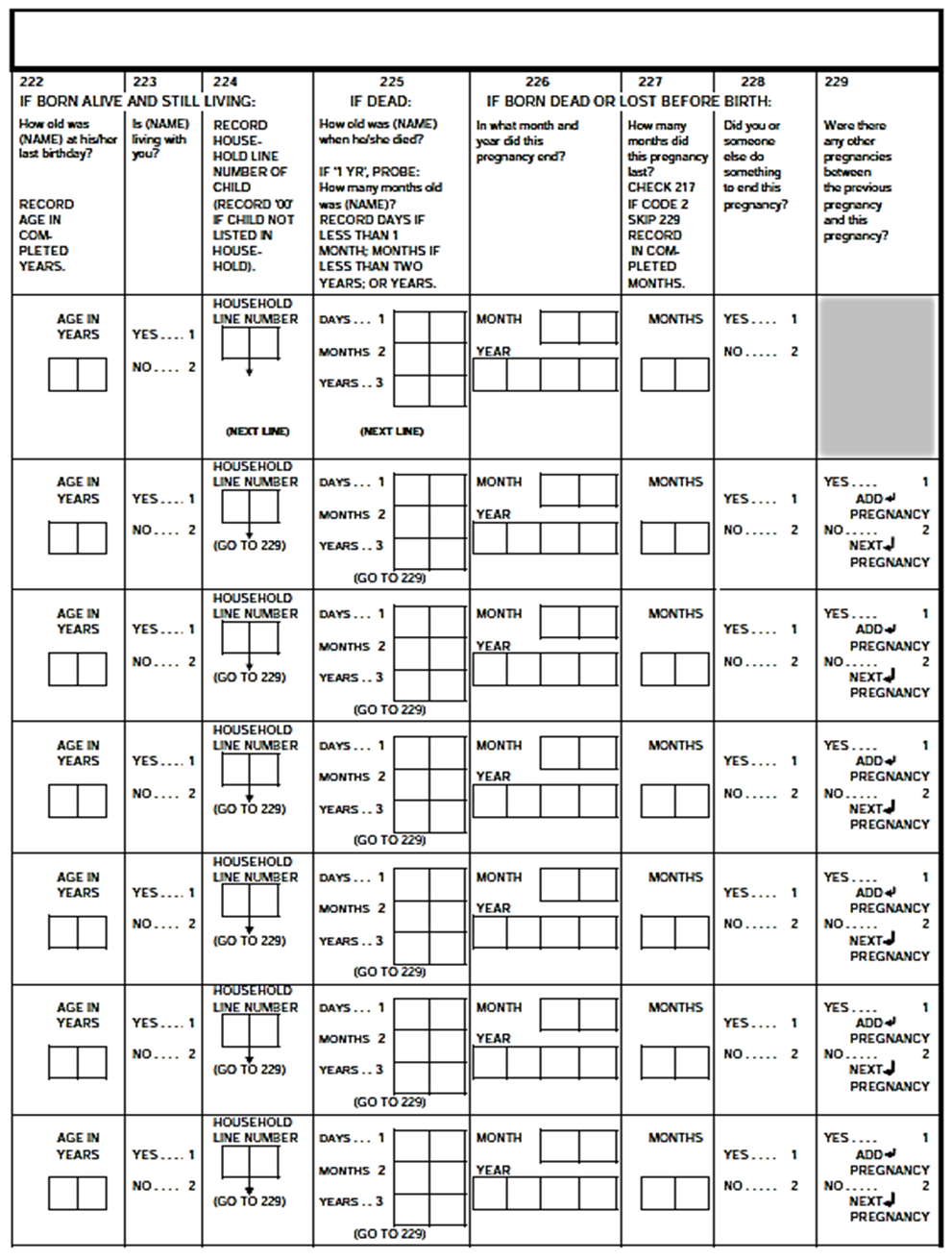
**

| **RH14.** | What was your age (in completed years) at the time of first birth? | 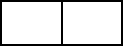 |  |
| --- | --- | --- | --- |
| **RH15.** | When you got pregnant, did you want to get pregnant at that time? | 1.Yes  2. No | **If yes Go to RH17** |
| **RH16.** | Did you want to have a baby later on or did not want any (more) children? | 1.Wanted later  2.Wanted no more |  |
| **RH17.** | What is the age of your youngest child? | 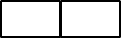 |  |
| **RH18.** | In your opinion what is the suitable age for having first pregnancy? | 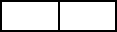 |  |
| **RH19.** | In your opinion after what age, a woman should not go for further pregnancies? | **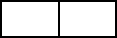** |  |
| **RH20.** | For better health of mother and infant, after giving birth how long a woman should wait to plan next pregnancy? (in months) | 1.At least 24 months  2.36 months  3.No longer than 5 years  96.Others(specify)________  98. Don’t know |  |
| **RH21.** | What could be the benefits of spacing births to mothers?  **( multiple response)** | 1.Reduces risk of pregnancy complications  2. Healthy recovery of mother for next preganancy  3. Increase chances of delivering a healthy baby  4. Sufficient time for breast feeding  5. Reduces risk of breast cancer  96. Any other __________  98. Don’t Know  99. Don’t Remember |  |
| **RH22.** | What could be the benefits of spacing births to a newborn?  **( multiple response)** | 1. Good health of newborn  2.Better care of neonate  3. Adequate duartion of breast  feeding  3.More care is provided by mother  4. Incraesed bonding between baby and mother  96. Any other __________  98. Don’t Know  99. Don’t Remember |  |
| **RH23.** | What could be the benefits of spacing births to the family?  **( multiple response)** | 1.Family welfare  2.Self satisfaction of husnband and wife due to family welfare  3.Provides an opportunity to father to strengthen economically to afford next child  96. Any other __________  98. Don’t Know  99. Don’t Remember |  |

| **Section E: Contraceptive Use** | | | **Response** |
| --- | --- | --- | --- |
| **CU1.** | Now I would like to talk about family planning - the various ways or methods that a couple can use to delay or avoid a pregnancy. Have you ever heard of (METHOD)?  FOR EACH METHOD WITH CODE 1 CIRCLED IN CU1, ASK CUA1. | | CU1A.Have you ever used the (METHOD)? |
| **CU1.1** | Female Sterilization. PROBE: Women can have an operation to avoid having any more children. | 1.Yes ( if no , skip the question about usage)  2.No | CUA1.1  Have you ever had an operation to avoid having any more pregnancies?  1.Yes  2.No |
| **CU1.2** | Male Sterilization. PROBE: Men can have an operation to Avoid having any more children. | 1.Yes  2.No | CUA1.2  Has your husband ever had an operation to avoid any more pregnancies?  1.Yes  2.No |
| **CU1.3** | IUD. PROBE: Women can have a loop or coil placed inside them by a doctor or a nurse. | 1.Yes  2.No | CUA1.3  1.Yes  2.No |
| **CU1.4** | Injectables. PROBE: Women can have an injection by a health provider that stops them from becoming pregnant for one or more months. | 1.Yes  2.No | CUA1.4  1.Yes  2.No |
| **CU1.5** | Implants. PROBE: Women can have one or more small rods placed in their upper arm by a doctor or nurse which can Prevent pregnancy for one or more years. | 1.Yes  2.No | CUA1.5  1.Yes  2.No |
| **CU1.6** | Pill. PROBE: Women can take a pill every day to avoid becoming pregnant | 1.Yes  2.No | CUA1.6  1.Yes  2.No |
| **CU1.7** | Emergency Contraception. PROBE: As an emergency measure, within three days after they have unprotected sexual intercourse, women can take special pills to prevent pregnancy? | 1.Yes  2.No | CUA1.7  1.Yes  2.No |
| **CU1.8** | Condom. PROBE: Men can put a rubber sheath on their penis Before sexual intercourse. | 1.Yes  2.No | CUA1.8  1.Yes  2.No |
| **CU1.9** | Standard Days Method. PROBE: A Woman uses a string of colored beads to know the days she can get pregnant. On the days she can get pregnant, they uses a condom or does not have sexual intercourse. | 1.Yes  2.No | CUA1.9  1.Yes  2.No |
| **CU1.10** | Lactational Amenorrhea Method (LAM) | 1.Yes  2.No | CUA1.10  1.Yes  2.No |
| **CU1.11** | Rhythm Method. PROBE: Every month that a woman is sexually active she can avoid pregnancy by not having sexual intercourse on the days of the month she is most likely to get Pregnant | 1.Yes  2.No | CUA1.11  1.Yes  2.No |
| **CU1.12** | Withdrawal. PROBE: Men can be careful and pull out before climax. | 1.Yes  2.No | CUA1.12  1.Yes  2.No |

| **CU2.** | Have said **YES** to any method in CU1A (**EVER USED** family planning) | 1.Yes  2.No | **If No, go to CU16** | |
| --- | --- | --- | --- | --- |
| **CU3.** | What method did you use? | **Last Method used** |  | |
| **CU3A.** | What year did you start it? How many years (completed years) did you take it? | Year of start  Month of start  Total duration  (months) |  | |
| **CU3B.** | Please mention the last 2 methods if more different methods used | 1.Female Sterilization.  2.IUD  3.Injectable  4.Implants  5.Pill  6.Condom  7.Standard Days Method  8.Lactational Amen Method  9.Withdrawl  10.Rhythm Method  96.Others |  | |
| **CU4.** | What was your age when you first started to use a contraceptive method? in completed years | 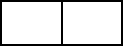 |  | |
| **CU5.** | How many living children you had when you first started using a contraceptive method? | 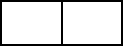 |  | |
| **CU6.** | Are you currently using last method to delay or avoid getting pregnant? | 1.Yes  2.No | **If No go to CU6B.** | |
| **CU6A.** | If yes for how many months you are regularly using that last method (without any discontinuation)? | 1. more than 6 months  2. 6 months | **GO TO CU7** | |
| **CU6B.** | If no for how long have you not using any method of contraception? | 1. more than 6 months  2. 6 months | **IF NO OR DISCONTINUED USER SKIP CU7** | |
| **CU7.** | Would you say that using contraception is mainly your decision, mainly your husband's decision, or did you both decide together? | 1.Mainly respondent  2.Mainly husband  3.Joint decision  96.Others_________________  (specify) |  | |
| **CU8.** | When started using your last method where did you get it at that time? | 1. Govt. Hospital/RHSA  2. Rural Health Centre  3. Family Welfare centre/FWW/ MCH  4.Dispensory/pharmacy  5. Mobile service camp/ unit  6. Lady health worker (LHW)  7.Lady Health Visitor (LHV)  8. Basic health unit  9.Male mobilizer  10.FWA (Family Welfare assistant)  11.Community Health Worker (CHW AMAN)  96.Other_____ (specify) |  | |
| **CU9.** | At that time, were you told about the side effects or problems you might have with the method? | 1. Yes  2. No |  | |
| **CU10.** | Were you told what to do if you experienced side effects or problems? | 1. Yes  2. No |  | |
| **CU11.** | Have you ever experienced side effects with your last family planning method used? | 1. Yes  2. No | **If No, go to CU16** | |
| **CU12.** | What major side effects did you experience**?**  **(Take multiple responses)** | 1. Headache.  2. Nausea/dizziness  3. Excessive bleeding  4. Spotting  5. Irregular menses/no  Menses  7. Depression  96.others ___________  99. Don’t remember |  | |
| **CU13.** | Did you seek any kind of treatment or medical advice for the side effects? | 1. Yes  2. No | **If No go to CU15** | |
| **CU14.** | From whom did you receive treatment? | **Public Sector**  1.Govt. Hospital/RHSC  2.Rural health centre  3.Family welfare centre/ FWW  4.MCH  5.Dispensery  6.Mobile service camp  7.Lady health worker  8. Lady Health visitor.  9.Basic health unit  10.Male mobilizer  11.FWA  96.Other Public________________  **Private/NGO Medical Sector**  12.Pharmacy, chemists  13.Private doctor  14.Homeopath  15. Dispenser/compounder  96.Other Private Medical _________specify)  **Other Source**  16.Shop(not pharmacy or dispensery)  17.Friend/relative  18.Hakim  19.Dai, Trad Birth Attendant .  96.Other _________ (specify)  98.Don't Know |  | |
| **CU15.** | Why you did not seek any treatment for side effects of contraceptive methods? | 1. Not necessary 2. Costs too much 3. Too far 4. No transport 5. No one to go with 6. Service not good 7. No time to go 8. Did not know where to go 9. Lady doctor was not available 10. Long waiting time 11. Not allowed to go   96. Others______ specify |  | |
| **CU16.** | Are you satisfied with the current method which you are using? | 1.Yes  2.No |  | |
| **CU17.** | Do you want a more effective method? | 1.Yes  2.No | **IF YES GO TO CU17A AND SKIP CU17B.**  **IF NO GO TO CU17B.** | |
| **CU17A.** | If yes then why? |  |  | |
| **CU17B.** | If No then why? |  |  | |
| **CU18.** | Did you ever get pregnant while using a method? | 1.Yes  2.No | **IF NO SKIP CU18A.** | |
| **CU18A.** | If yes what could be the reason? | 1.Did not know how to use the method  2.Missed a dose  3.Method did not work for me  96.Any other |  | |
| **CU19.** | At the time, When you obtained your last method were you told about other methods that you could use? | 1. Yes  2. No  99. Don’t remember |  | |
| **CU20.** | From where did you obtain your last method last time? | **Public Sector**  1.Govt. Hospital/RHSC  2.Rural health centre  3.Family welfare centre/ FWW  4.MCH  5.Dispensery  6.Mobile service camp  7.Lady health worker  8. Lady Health visitor.  9.Basic health unit  10.Male mobilizer  11.FWA  96. Any other Public________________  (specify)  **Private/NGO Medical Sector**  12.Pharmacy, chemists  13.Private doctor  14.Homeopath  15. Dispenser/compounder.  16. Aman health worker  96.Other Private Medical _________(specify)  **Other Source**  1*7.*Shop(not pharmacy or dispensery)  18.Friend/relative  19.Hakim  20.Dai, Trad Birth Attendant .  96. Any other _________ (specify)  98.Don't Know  99. Don’t Remember |  | |
| **CU21.** | In the last 6 months, did a health worker visit you? | 1. Yes  2. No | **If “No” go to**  **CU 24** | |
| **CU22.** | Which health organization was she from? | 1. Government 2. Sukh Aman 3. Both   96. Any other (specify)  98.Don't Know  99. Don’t Remember |  | |
| **CU23.** | What information did you receive from her? | 1. Mother and child health  2. Family planning  3.Contraceptive supplies  4.Referred to health/FP facility  5.Treatment of side effects  6.TT /child Vaccination  7.Treatment of minor ailments  8. information about tele health services  9.Information about Family life education  10. Information about community group discussions regarding Family Life Education an Family Planning  96. Any other (specify)  98.Don't Know  99. Don’t Remember |  | |
| **CU23a.** | Are you satisfied with services provided by the health worker (encircle each option, if satisfied)   1. Aman CHWs 2. LHWs   **(Circle those which apply)** | 1. Information regarding maternal and child care  2. Child immunization services  3. Counselling regarding family planning  4. Supply of family planning commodities (pills/condoms)  5. Treatment/advice for contraceptive side-effects  6. Referral to health facility /FP facility  7.Treatment of minor ailments  8. Duration of visit  9. Provision of medicines  10. Information regarding breast feeding  11.Information regarding postpartum care  12.Information regarding post-abortion care  13. Maternal and child nutrition  14.Attitude of CHW towards your queries  15. Accompanied you to a health facility  16.Checked BP  17.Checked weight of the mother  18.Checked the weight of children under three  96. Any other (specify)  98.Don't Know  99. Don’t Remember | Aman CHW | LHW |
| **CU24.** | In the last 6 months, have you visited a health facility for care of yourself (or your children)? | 1. Yes  2. No | **IF NO GO TO CU25** | |
| **CU25.** | If no what was reason for not visiting health care facility?  **( multiple response)** | 1.Services provided at their door steps  2. Preferred to go to other service providers  3.Due to undesirable location  4. No need to visit the centre  5. Unaware about the service available at the centre  6.Wanted more children  96. Any other (specify)  98.Don't Know  99. Don’t Remember |  | |

**Questions related to assessing the knowledge regarding modern contraceptive and its use.**

| **CONDOMS (CD)** | | | |
| --- | --- | --- | --- |
| **CD1** | Do you think pregnancy can occur while using a condom? | 1. Yes  2. No.  98. Don’t know  96. others (specify)______________ | **If No or don’t Know, go to CD3** |
| **CD2** | If yes why?  **(take multiple responses)** | 1. If put on wrongly 2. Spilling semen over the vaginal opening while removing 3. if condom ruptures while being used   98. Don’t know  99. Don’t remember  96. Others (Specify) ------------ |  |
| **CD3** | In your opinion How effective is the condom for preventing pregnancy? | 1. Very effective  2. Effective  3. Not very effective  4. Not effective  98. Don’t know  99. Don’t remember  96. Others(Specify)------------ |  |
| **CD4** | Are condoms easily available to the people of this area? | 1. Yes  2. No.  98. Don’t know  99. Don’t remember  96. Others(Specify)------------ |  |
| **CD5** | In your opinion, are condoms easily affordable to the people of this area? | 1. Yes  2. No.  98. Don’t know  99. Don’t remember  99. NA  96. Others(Specify)------------ |  |
| **CD6** | What is the one main reason for liking this method? | 1.Low cost  2.Easily available  3.Prevents STDs  4.Easy to use  5.No side effects  98. Don’t know  99. Don’t remember  96. Others(Specify)  ------------ |  |
| **CD7** | What is the one main thing which you don’t like about this method?  **(Take one response)** | 1. May cause allergy to man 2. May cause allergy to Woman 3. Make sex less enjoyable 4. Embarrassing to ask for condoms 5. Difficult to dispose off 6. Don’t like it   98. Don’t know  99. Don’t remember  96. Others (Specify)_________ |  |
| **CONTRACEPTIVE PILLS (OCP)** | | | |
| **OCP1.** | How are OCPs taken? | 1.One pill to be taken every day  2.Pills are taken at the time of intercourse  3.Once a week  96. Others (Specify) ------------  98. Don’t know  99. Don’t remember |  |
| **OCP2.** | Do you think pregnancy can occur while using pills? | 1. Yes  2. No.  98. Don’t know  96. others (specify)______________ |  |
| **OCP3.** | In your opinion, how effective are OCPs for preventing pregnancy? | 1. Very effective  2. Effective  3. Not very effective  4. Not effective  98. Don’t know  99. Don’t remember  96. Others(Specify)------------ |  |
| **OCP4.** | In your opinion, are OCPs easily available in your area? | 1. Yes  2. No.  98. Don’t know  99. Don’t remember  96. Others(Specify)------------ |  |
| **OCP5.** | In your opinion, are OCPs easily affordable for you? | 1. Yes  2. No  98. Don’t know  99. Don’t remember  96. Others(Specify)------------ |  |
| **OCP6.** | What is the ONE main reason for liking this method OCPs? (**Take one response only)** | 1.Low cost  2.Easily available  3.No side effects  4.Woman can use it without husband’s knowledge  5.Easy to use  6.Non-interference with  sexual intercourse  7.Temporary method  96. Others (Specify)____________  98. Don’t know  99. Don’t remember |  |
| **OCP7.** | What is the one main thing which you don’t like about this method? | 1.Results in heavy bleeding  2.Results in break through bleeding  3.Results in dysmenhorrhea  4.Results in weight gain  5.Results in nausea vomiting  6.Has to be taken every day  7.Don’t know about the method  96.Others(Specify)__________  98. Don’t know  99. Don’t remember |  |
| **Injection** | | | |
| **INJ1** | In your opinion how frequently are FP injections taken? | 1. Taken every 2 -3 months 2. Every month   98. Don’t know  99. Don’t remember  96. Others(Specify)----------- |  |
| **INJ2** | In your opinion How effective are injections for preventing the pregnancy? | 1. Very effective  2. Effective  3. Not very effective  4. Not effective  96. Others(Specify)__________  98. Don’t know  99. Don’t remember |  |
| **INJ3.** | Do you think pregnancy can occur while using injection? | 1. Yes  2. No.  98. Don’t know  96. others (specify)______________ |  |
| **INJ4.** | Are injections easily available in your area? | 1. Yes  2. No.  96. Others(Specify)__________  98. Don’t know  99. Don’t remember |  |
| **INJ5.** | Are injections easily affordable for you? | 1. Yes  2. No.  98. Don’t know  99. Don’t remember  96. Others(Specify)------------ |  |
| **INJ6.** | What is the ONE main reason for liking this method ?**(Take one response)** | 1.Doesn’t have to be taken every day  2.Can be used by nursing mothers  3.Provides contraception for  2-3 months  4.Can be stopped any time  5.Can be used secretly  1.Easily available  2.Low cost  3.No interference with sex  98. Don’t know  99. Don’t remember  96. Others (Specify  ________________ |  |
| **INJ7** | What is the one main thing which you don’t like about this method?  **(Take one response)** | 1.Difficulty in remembering the date for subsequent dose  2.Have to repeat the injection every 2 to 3 months  3.Results in heavy menstrual bleeding  4.Results in break through bleeding  5.Results in dysmenorrheal  6.Results in weight gain  7.Results in amenorrhea  8.Results in nausea/vomiting  98. Don’t know  99. Don’t remember  96. Others (Specify)-------- |  |
| **IUCD (IUD)** | | | |
| **IUD1** | Where is IUCDs placed in the body? | 1. IUCD is placed in uterus 2. IUCDs is placed internally (intravginally) 3. IUCDs is placed inside the abdomen   98. Don’t know  99. Don’t remember  96. Others (Specify)  ___________ |  |
| **IUD2** | What is the duration of contraception provided by IUCD? | 1. 5 years 2. 10 years 3. 2-3 years 4. The woman can have an IUCD with no time limitation   98. Don’t know  99. Don’t remember  96. Others (Specify)  ___________ |  |
| **IUD3.** | Do you think pregnancy can occur while using an IUCD? | 1. Yes  2. No.  98. Don’t know  96. others (specify)______________ |  |
| **IUD4.** | In your opinion, how effective are IUCDs for preventing pregnancy? | 1. Very effective  2. Effective  3. Not very effective  4. Not effective  98. Don’t know  99. Don’t remember  96. Others(Specify)------------ |  |
| **IUD5.** | Are IUCDs easily available in your area? | 1. Yes  2. No.  98. Don’t know  99. Don’t remember  96. Others(Specify)------------ |  |
| **IUD6.** | Are IUCDs easily affordable in your area? | 1. Yes  2. No.  98. Don’t know  998. Don’t remember  99. NA  96. Others(Specify)------------ |  |
| **IUD7.** | How frequently does the woman have to check the thread of IUCD? | 1. Once a month after each menstrual cycle 2. Once a week 3. Every 3 months 4. Whenever she wants   98. Don’t know  99. Don’t remember  96. Others(Specify)------------ |  |
| **IUD8.** | What is the ONE main reason for liking this method ? **(Take one response)** | 1. Provides spacing for 2 years -5 years 2. Can be used by nursing mothers 3. Can be removed anytime 4. No fear of forgetting the dose like pills and injection 5. No side effects 6. No interference with sex   98. Don’t know  99. Don’t remember  96. Others(Specify)------------ |  |
| **IUD9.** | What is the one main thing which you don’t like about this method? (Take one response) | 1. Results in heavy bleeding 2. Results in break through bleeding 3. Results in dysmenorrhea 4. Results in dyspareunia 5. Hurts husband at the time of intercourse 6. Dislodgment from the uterine cavity 7. Results in infection/swelling 8. Results in cancer of the uterus 9. It goes into the muscles of uterus   96.Others(Specify)________  98. Don’t know  99. Don’t remember |  |
| **Implant (IP)** | | | |
| 1. **IP1** | 1. What is the site for implant insertion? | 1. Sticks are inserted in left upper arm  2. Inside uterus  98. Don’t know  99. Don’t remember  96. Others (Specify)----- |  |
| **IP2** | 1. What is the duration of contraception provided by implant? | 1. 1. 5 years 2. 2. 10 years 3. 3. 3 months 4. 4. 2-3 years 5. 98. Don’t know 6. 99.Don’t remember 7. 96. Others (Specify)----- |  |
| 1. **IP3** | 1. In your opinion how effective is implant for preventing pregnancy? | 1. Very effective  2. Effective  3. Not very effective  4. Not effective  98. Don’t know  99. Don’t remember  96. Others(Specify)------------ |  |
| 1. **IP4.** | Do you think pregnancy can occur while using implant? | 1. Yes  2. No.  98. Don’t know  96. others (specify)______________ |  |
| 1. **IP5.** | 1. Is implant easily available in your area? | 1. Yes  2. No.  98. Don’t know  99. Don’t remember  96. Others(Specify)------------ |  |
| 1. **IP6.** | 1. Is implant easily affordable in your area? | 1. Yes  2. No.  98. Don’t know  99. Don’t remember  96. Others(Specify)------------ |  |
| 1. **IP7.** | 1. What is the ONE main reason for liking this method for spacing birth?   **(Take one response only)** | 1.Provides contraception for 5 years  2.Can be used by nursing Mothers  3.No side effects  98. Don’t know  99. Don’t remember  96. Others(Specify)------------ |  |
| **IP8.** | What is the one main thing which you don’t like about this method?  **(Take one response)** | 1. Results in heavy bleeding 2. Results in breakthrough bleeding 3. Results in dysmenorrhea 4. Dislodgment from the place of insertion 5. Results in infection/swelling   6. Vaginal discharge  98. Don’t know  99. Don’t remember  96. Others(Specify)------------ |  |

| **Section F: Discontinuation of Contraceptive Use** | | | **Response** |
| --- | --- | --- | --- |
| **Now I would like to ask questions to the ever users who discontinued using a modern contraceptive method since more than 6 months.** | | | |
| **DC1** | Since what month and year did you stop using (LAST METHOD)? | **Month**  **Year** |  |
| **DC2** | What was the method which you discontinued? | 01.Injectable  02. Implants.  03.Pill  04.Female Condom  05. IUD  96.Others Specify |  |
| **DC3** | What were the reasons for discontinuing the last method? | 01.Lack of access/too far  02.Preferred method not available  03.Inconvenient to use  04.Interferes with body’s normal  Process  05 Side Effects  06. Infrequent sex.  07.Not having Sex  08.Up to GOD  09.Breast feeding  10. Fear of side effects.  11. Husband opposed  12. Religious prohibition  13.Friend’s opposition/Neighbourer’s opposition/Relatives  opposition  14.Wanted to become pregnant  96. Others specify |  |
| **DC4.** | **What was the main reason of discontinuing the last method?** | See methods below and answer accordingly |  |
| **DC4.1** | What was the main reason of discontinuing Condom? | 01.May cause allergy to man  02.May cause allergy to Woman  03.Make sex less enjoyable  04.Embarrassing to ask for condoms  05.Difficult to dispose off  06. Don’t like it  98. Don’t know  99. Don’t remember  96. Others (Specify)_________ |  |
| **DC4.2** | What was the main reason of discontinuing Contraceptive Pill? | 1.Results in heavy bleeding  2.Results in break through bleeding  3.Results in dysmenhorrhea  4.Results in weight gain  5.Results in nausea vomiting  6.Has to be taken every day  7.Don’t know about the method  96.Others(Specify)__________  98. Don’t know  99. Don’t remember |  |
| **DC4.3** | What was the main reason of discontinuing Injection? | 1.Difficulty in remembering the date for subsequent dose  2.Have to repeat the injection every 2 to 3 months  3.Results in heavy menstrual bleeding  4.Results in break through bleeding  5.Results in dysmenorrheal  6.Results in weight gain  7.Results in amenorrhea  8.Results in nausea/vomiting  98. Don’t know  99. Don’t remember  96. Others (Specify)-------- |  |
| **DC4.4** | What was the main reason of discontinuing IUCD? | 1. Results in heavy bleeding 2. Results in break through bleeding 3. Results in dysmenorrhea 4. Results in dyspareunia 5. Hurts husband at the time of intercourse 6. Dislodgment from the uterine cavity 7. Results in infection/swelling 8. Results in cancer of the uterus 9. It goes into the muscles of uterus   96.Others(Specify)________  98. Don’t know  99. Don’t remember |  |
| **DC4.5** | What was the main reason of discontinuing implant? | 01.Results in heavy bleeding  02.Results in breakthrough bleeding  03.Results in dysmenorrhea  04.Dislodgment from the place of insertion  05.Results in infection/swelling  06.Vaginal discharge  98. Don’t know  99. Don’t remember  96. Others(Specify)------------ |  |
| **DC5.** | Would you say that discontinuation of contraception is mainly your decision; mainly your husband’s decision, or did you both decided together? | 01 Mainly respondent  02 Mainly husband  03 Joint decision  96 others |  |
| **DC6.** | Do you prefer any traditional method over modern method? | 01.Yes  02.No |  |
| **DC7.** | Do you think modern method cost too much to continue its use in future? | 01.Yes  02.No |  |
| **DC8.** | Are the sources (outlets) either private or public from where you received the modern methods are too far? | 01.Yes  02.No |  |
| **DC9.** | Do you think is it right to discontinue a method for time being and then recontinue it? | 01.Yes  02.No |  |
| **DC10.** | Would you go for a modern contraceptive method in the future again in order to space birth? | 01.Yes  02.No |  |
